# An oncolytic virus–delivered TGFβ inhibitor overcomes the immunosuppressive tumor microenvironment

**DOI:** 10.1084/jem.20230053

**Published:** 2023-08-08

**Authors:** Kristin DePeaux, Dayana B. Rivadeneira, Konstantinos Lontos, Victoria G. Dean, William G. Gunn, McLane J. Watson, Tianhong Yao, Drew Wilfahrt, Cynthia Hinck, Lukasz Wieteska, Stephen H. Thorne, Andrew P. Hinck, Greg M. Delgoffe

**Affiliations:** 1 Department of Immunology, University of Pittsburgh School of Medicine, Pittsburgh, PA, USA; 2 Stem Cell Transplantation and Cellular Therapy Center, The University of Texas, MD Anderson Cancer Center, Houston, TX, USA; 3 Department of Metabolism and Nutritional Programming, Van Andel Institute, Grand Rapids, MI, USA; 4 Department of Structural Biology, University of Pittsburgh School of Medicine, Pittsburgh, PA, USA; 5 Kalivir Immunotherapeutics, Pittsburgh, PA, USA

## Abstract

While checkpoint blockade immunotherapies have widespread success, they rely on a responsive immune infiltrate; as such, treatments enhancing immune infiltration and preventing immunosuppression are of critical need. We previously generated αPD-1 resistant variants of the murine HNSCC model MEER. While entirely αPD-1 resistant, these tumors regress after single dose of oncolytic vaccinia virus (VV). We then generated a VV-resistant MEER line to dissect the immunologic features of sensitive and resistant tumors. While treatment of both tumor types induced immune infiltration and IFNγ, we found a defining feature of resistance was elevation of immunosuppressive cytokines like TGFβ, which blunted IFNγ signaling, especially in regulatory T cells. We engineered VV to express a genetically encoded TGFβRII inhibitor. Inhibitor-expressing VV produced regressions in resistant tumor models and showed impressive synergy with checkpoint blockade. Importantly, tumor-specific, viral delivery of TGFβ inhibition had no toxicities associated with systemic TGFβ/TGFβR inhibition. Our data suggest that aside from stimulating immune infiltration, oncolytic viruses are attractive means to deliver agents to limit immunosuppression in cancer.

## Teaser

Oncolytic viruses induce tumor lysis and inflame the tumor microenvironment but do not relieve immunosuppressive signals. Engineering oncolytic vaccinia virus to express a potent TGFβR inhibitor results in local neutralization of TGFβ, increased T_reg_ cell fragility, and superior therapeutic response.

## Graphical Abstract

**Figure:**
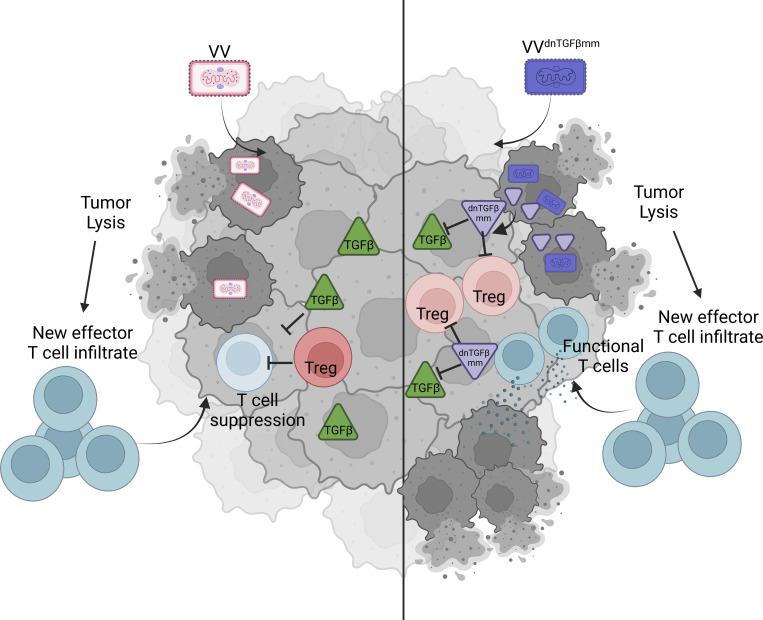

## Introduction

As cancer progresses, it establishes an immunosuppressive tumor microenvironment (TME) that alters local stromal and immune cells to prevent immune infiltration, recognition, and function. These include physical barriers to infiltration, alterations in the local metabolic milieu, the recruitment of immunosuppressive cell types, and elevation of soluble factors that can dampen immunity (DePeaux and Delgoffe, 2021). Many of these features have been shown to promote tumor progression at the steady state, but also are associated with resistance to immunotherapies like checkpoint blockade, which utilizes monoclonal antibodies to block inhibitory receptors like PD-1 on the surface of infiltrating T cells.

Indeed, checkpoint blockade immunotherapies have had widespread success in a variety of solid tumors, prolonging the lives of millions of patients. However, these therapies rely on the presence of a pre-existing immune infiltrate, and in patients that carry immunologically inert tumors, checkpoint blockade has shown little success. Thus, there remains a critical need for treatment modalities that can promote immune infiltration as well as limit suppression within the TME.

Oncolytic viruses (OVs) preferentially infect tumor cells and can be utilized for cancer therapy. These viruses, by natural tropisms and engineered selectivity, replicate in tumor cells leading to tumor lysis, release of pathogen-associated molecular patterns and damage-associated molecular patterns, and ultimately T cell priming with tumor and viral antigen (Bommareddy et al., 2018). The adaptive immune response is stimulated in response to the patient’s own tumor neo-antigens, acting essentially as a patient-specific vaccination (Zamarin et al., 2014; Oh et al., 2017; Fend et al., 2015). This immune-stimulatory action of OVs has the potential to inflame the TME and initiate new antitumor immunity. Currently, there is only one FDA-approved OV in the US, talimogene laherparepvec (T-VEC, Imlygic), an oncolytic herpesvirus, approved for use in advanced melanoma (Andtbacka et al., 2015). Recently, there has been interest in combining checkpoint blockade with OV therapy (Ribas et al., 2017; Nakao et al., 2020; Hwang et al., 2020), and these combinations, along with preclinical studies with other types of OVs, have had preclinical promise. However, there have been difficulties translating these findings to clinical success, demonstrating that there is still much to understand about their mechanism of action.

A key defining feature of OVs is their ability to be engineered not only to promote tumor selectivity but also to deliver gene therapy to the TME. T-VEC, for instance, delivers the gene for GM-CSF to the tumor (Andtbacka et al., 2015) to support APC recruitment. But these genetic payloads need not be immunologic in nature; for instance, our group has demonstrated that metabolic support can be delivered to new tumor infiltrate by encoding adipokines like leptin into the virus (Rivadeneira et al., 2019). While most OVs in the clinic are designed to deliver immune stimulation, encoding cytokines like IL-2, IL-12, etc., OVs, in general, promote robust immune infiltration on their own (Rivadeneira et al., 2019). Thus, genetic payload rationally designed to augment immunity through other mechanisms may be more efficacious.

While many OV studies report the induction of cytotoxic CD8^+^ T cells after treatment, the role of immunoregulatory factors is less apparent in OV therapy despite controlling resistance mechanisms in the TME. Key among these resistance mechanisms is the recruitment of regulatory T (T_reg_) cells to the TME and the heightened concentration of inhibitory cytokines like TGFβ. T_reg_ cells, among other factors present in the TME, including soluble, cellular, and structural factors, can prevent immune infiltration and activity, and consequently immunotherapy efficacy (DePeaux and Delgoffe, 2021). Thus, we sought to study the immunoregulatory mechanisms associated with OV resistance and engineer OVs to neutralize these inhibitory factors.

In this study, we used serial in vivo passaging of a tumor model sensitive to oncolytic vaccinia virus (VV) treatment to generate paired isogenic tumor cell lines resistant or sensitive to therapy. This allowed direct comparison for deep understanding of the immunologic mechanisms underlying OV-responsive tumors. Analysis of the infiltrate from these various tumor models revealed that while tumors treated with OVs showed infiltration of new T cells, a defining feature within the TME of resistant tumors was the phenotypic stability of T_reg_ cells, linked directly to increases in TGFβ production. We engineered an oncolytic vaccinia that expressed a potent TGFβ inhibitor and found that mice bearing resistant tumors exhibited dramatic responses when treated with this agent, which could be extended to synergy with checkpoint blockade therapy in an extremely aggressive melanoma model.

## Results

### Response to oncolytic vaccinia is not determined by viral kinetics or oncolysis

**Figure 1.**
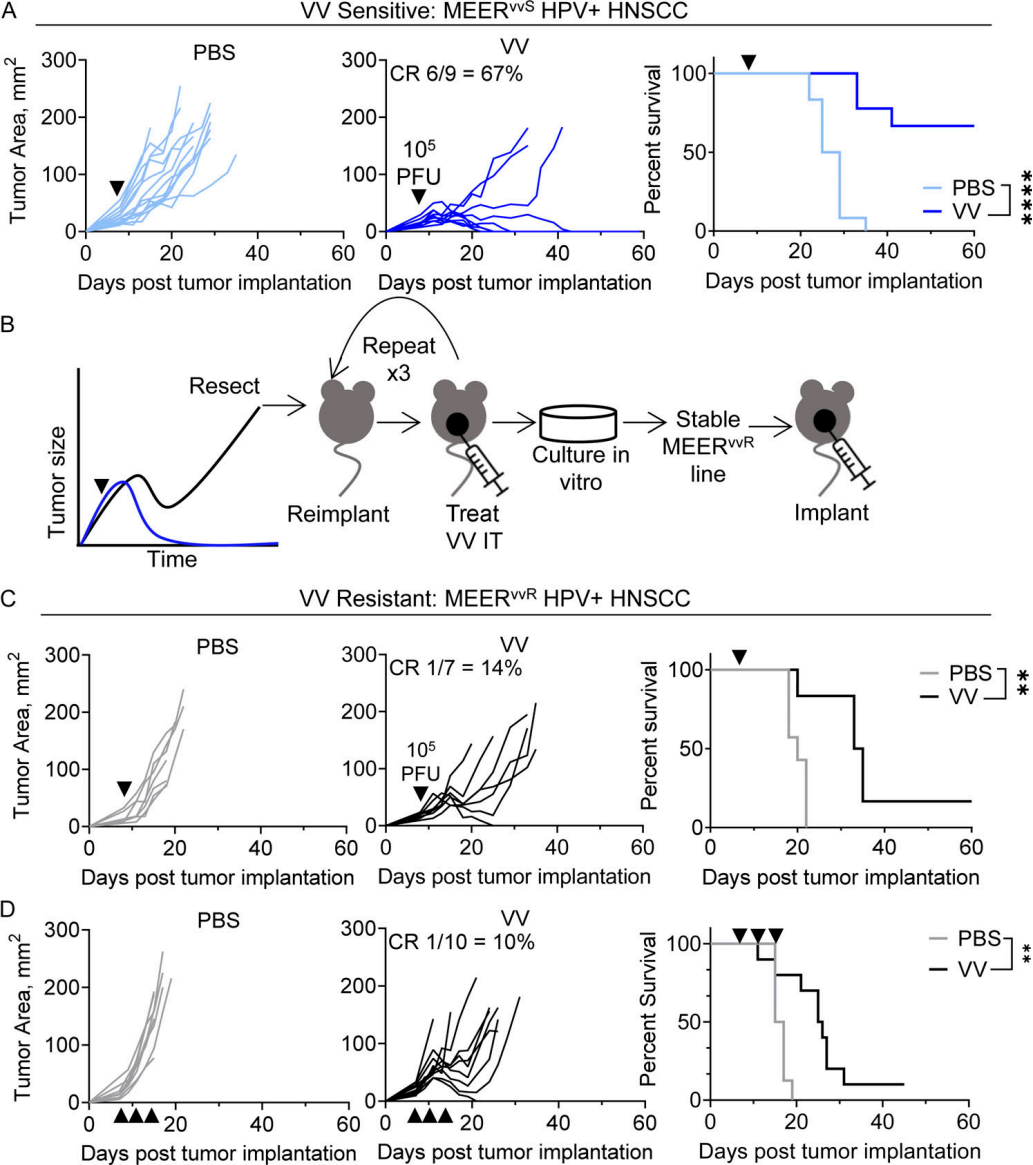
**Generation of paired tumor lines with differential response to OV immunotherapy. (A)** Tumor growth (left, middle) and survival (right) of C57BL/6 mice implanted intradermally with MEER^vvS^ and, when tumors were ∼20 mm^2^, treated with a single IT injection of VV at 2.5 × 10^5^ PFU/mouse or PBS control (black arrowhead). Mice were sacrificed when tumors reached 15 mm in any direction. **(B)** Schematic depicting the generation of MEER^vvR^ from MEER^vvS^ cells. **(C)** As in A, but with MEER^vvR^. **(D)** As in C, but with repeated dosing. Mice were dosed three times with IT VV (2.5 × 10^5^ PFU/mouse) every 4 d, starting 7 d after implantation. Data represent three independent experiments (A, C, and D). Each line represents an individual mouse. **P < 0.01, ****P < 0.0001 by Mantel-Cox test (A, C, and D).

We sought to determine the immunologic mechanisms of resistance to OVs. To do this, we first identified a tumor model that responds to oncolytic vaccinia and subsequently developed a resistant line from it. The murine model of HPV+ (human papilloma virus) head and neck squamous cell carcinoma (HNSCC), MEER, a mouse oropharyngeal line transformed with h*RAS* and HPV E6/E7, is partially sensitive to anti-PD-1 therapy (Zandberg et al., 2021). Our lab has previously rendered this line αPD-1 resistant through serial passaging and treatment in mice (Zandberg et al., 2021). Mice bearing this checkpoint blockade–resistant MEER variant were treated with a single intratumoral (IT) dose (2.5 × 10^5^ PFU/mouse) of oncolytic vaccinia (VV, Western reserve strain). This oncolytic, “double deleted” vaccinia virus has a luciferase reporter, an insertion of GFP in the thymidine kinase (TK) locus, and a deletion of virulence growth factor, which make the virus more selective for replication in tumor cells (McCart et al., 2001). Remarkably, after a single IT dose of VV, we observed complete tumor clearances (complete responses, CR) in 67% of mice and a significant extension of survival compared with PBS IT injection control (Fig. 1 A). Thus, this aggressive model is remarkably sensitive to VV while being entirely resistant to αPD-1 therapy.

At this treatment dose, some mice did not experience tumor regression and ultimately succumbed to disease. One such tumor was resected from a treated animal and implanted into new mice. Once reaching 20 mm^2^, these tumors were treated with VV (IT, 2.5 × 10^6^ PFU/mouse) and monitored for response to therapy. Treatment-resistant tumors were again resected, and this process was repeated three times until a MEER derivative (termed MEER^vvR^) stably resistant to VV therapy was generated (Fig. 1, B and C). Treatment of MEER^vvR^ tumors now carried just a 14% CR rate, compared to 67% of the parental line, termed MEER^vvS^ going forward_._ As patients in the clinic are most commonly treated with multiple doses of OV, we next treated MEER^vvR^-bearing mice with a repeated dosing regimen to determine if they retained VV resistance. Mice bearing MEER^vvR^ tumors were treated IT with VV and given two additional doses at 4 and 8 d after the initial treatment. We found that with repeated dosing, the CR rate was almost exactly the same as with single-dose therapy, 10% compared to 14% (Fig. 1 D). Thus, MEER tumors maintain resistance to VV therapy even with multiple doses.

**Figure S1.**
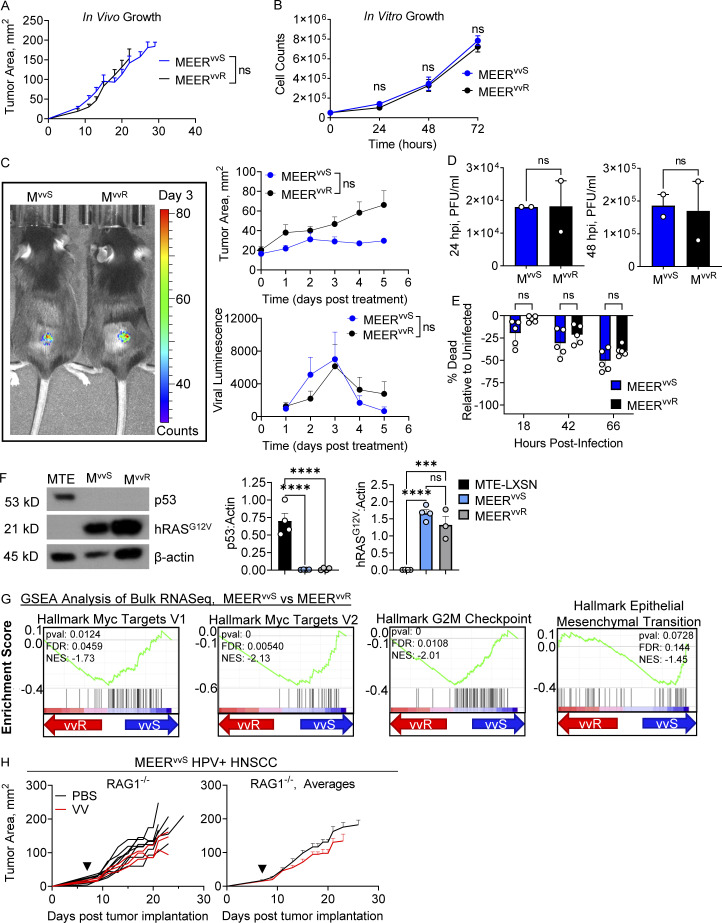
**Viral kinetics are unchanged between MEER^vvS^ and MEER^vvR^. (A)** Tumor growth of C57BL/6 mice implanted intradermally with MEER^vvS^ or MEER^vvR^ as in Fig. 1. **(B)** In vitro expansion of 50,000 MEER^vvS^ and MEER^vvR^ cells. **(C)** Quantified luminescence and tumor area of C57BL/6 mice implanted intradermally with MEER^vvS^ or MEER^vvR^ and treated with VV (2.5 × 10^5^ PFU/mouse), then every 24 h injected IP with luciferin and imaged. A representative image from day 3 after treatment is shown. **(D)** Viral titers of supernatants collected from MEER^vvS^ or MEER^vvR^ infected with VV at an MOI of 0.1 or mock infected for 2 h, washed, and replaced with fresh media. Titers were calculated by plaque assay on HeLa cells. **(E)** MEER^vvS^ and MEER^vvR^ infected as in D were stained with Zombie viability dye and run on the flow cytometer at the indicated times to observe viability. Percent viability normalized to mock-infected controls. **(F)** Representative image and quantification of Western blots of MEER^vvS^, MEER^vvR^, and MTE-LXSN cells cultured as in B then harvested for Western blot and stained for p53, hRAS^G12V^, and β-actin. **(G)** Enrichment plots of gene set enrichment analysis (GSEA) Hallmark analysis of MEER^vvS^ and MEER^vvR^ cultured as in B. FDR, false discovery rate; NES, normalized enrichment score. **(H)** Tumor growth of VV treated MEER^vvS^ tumors as in Fig. 1, but in *Rag1*^*−/−*^ mice. Data represent two (D–F) or three independent experiments (A–C, E, and H). Each point represents an individual mouse (A, C, and G) or technical replicate (B and D–F). ***P < 0.001, ****P < 0.0001 by two-way ANOVA with Tukey’s multiple comparison test (A–C and E), unpaired *T* test (D and F), or Mantel-Cox test (H). ns, non-significant. Error bars indicate SEMs. Source data are available for this figure: SourceData FS1.

Using these two lines, we then investigated potential causes for the differential response to VV. First, we determined that there were no baseline differences in tumor growth, either in vivo (Fig. S1 A) or in vitro (Fig. S1 B). Next, we used in vivo luciferase imaging to investigate differences in viral replication as a potential resistance mechanism. Mice bearing MEER^vvS^ or MEER^vvR^ were treated IT with 2.5 × 10^5^ PFU of VV, which contains a virally encoded luciferase, and imaged using the IVIS system every 24 h for 5 d after treatment. We found no significant differences in viral replication between the MEER^vvS^ and MEER^vvR^ tumors and found that viral replication peaked at day 3 (Fig. S1 C). Notably, there is no significant difference in tumor size during days 0–5 of treatment (Fig. S1 C); tumor growth does not diverge until days 7–10 after treatment (Fig. 1 A). Next, we tested how much active virus could be produced by the tumor lines. MEER^vvS^ and MEER^vvR^ were plated and infected with VV at a multiplicity of infection (MOI) of 0.1 and supernatant was harvested at 24 and 48 h. This supernatant was tittered on HeLa cells using a crystal violet plaque assay. No significant difference in virus production was observed (Fig. S1 D). We tested viral-induced cell death between the tumor lines. Again, MEER^vvS^ and MEER^vvR^ were plated and infected with VV at an MOI of 0.1. Viability was determined by flow cytometry over time (Fig. S1 E) with no significant differences at any tested timepoint. These data show that viral kinetics and oncolysis were unchanged by rendering the tumor VV resistant.

The original MEER line was generated by transforming murine tonsillar epithelial cells (MTE) with HPV16 E6/E7 and hRas (Hoover et al., 2007). To ensure that during the generation of resistance in these lines they did not lose these transgenes, we performed Western blot analysis of p53, which is targeted for proteasomal degradation by HPVE6/E7 and subsequently lost, and mutant hRAS^G12V^ in MEER^vvS^, MEER^vvR^, and MTE-LXSN, which are MTE cells containing the control empty vector (EV). We found that MTE-LXSN cells retained p53 expression that was lost in both MEER lines (Fig. S1 F). This showed that there was no loss of E6/E7 expression in either line, as treatment with a proteasomal inhibitor leads to the return of p53 in as little as 3 h (Hoover et al., 2007). The MEER lines also had no significant difference in hRAS^G12V^ expression which was not present in the MTE-LXSN cells (Fig. S1 F). We also performed bulk RNA sequencing (RNA-seq), and through gene set enrichment analysis found repression in MEER^vvS^ of Myc target genes, G2/M checkpoints, and epithelial to mesenchymal transition compared with MEER^vvR^ (Fig. S1 G). These changes suggest that while gaining resistance to VV therapy, the MEER^vvR^ line maintained its identity while acquiring several beneficial programs to generally support resistance.

We next sought to determine if adaptive immunity was required for sensitivity to VV therapy. To test this, we treated RAG1-deficient mice bearing MEER^vvS^ tumors with VV as in previous studies. We found no difference in tumor growth between PBS control and VV-treated tumors in this model (Fig. S1 H), suggesting that the adaptive immune response is critical for VV therapy and that the lytic effect of the virus is not sufficient for tumor clearance. Taken together, these data suggest that CRs to OVs observed in MEER^vvS^ were due to an effect on the adaptive immune system, not an inherent sensitivity to oncolysis or enhanced viral spread.

### MEER^vvR^ tumors contain higher concentrations of TGFβ and more stable T_reg_ cells

**Figure 2.**
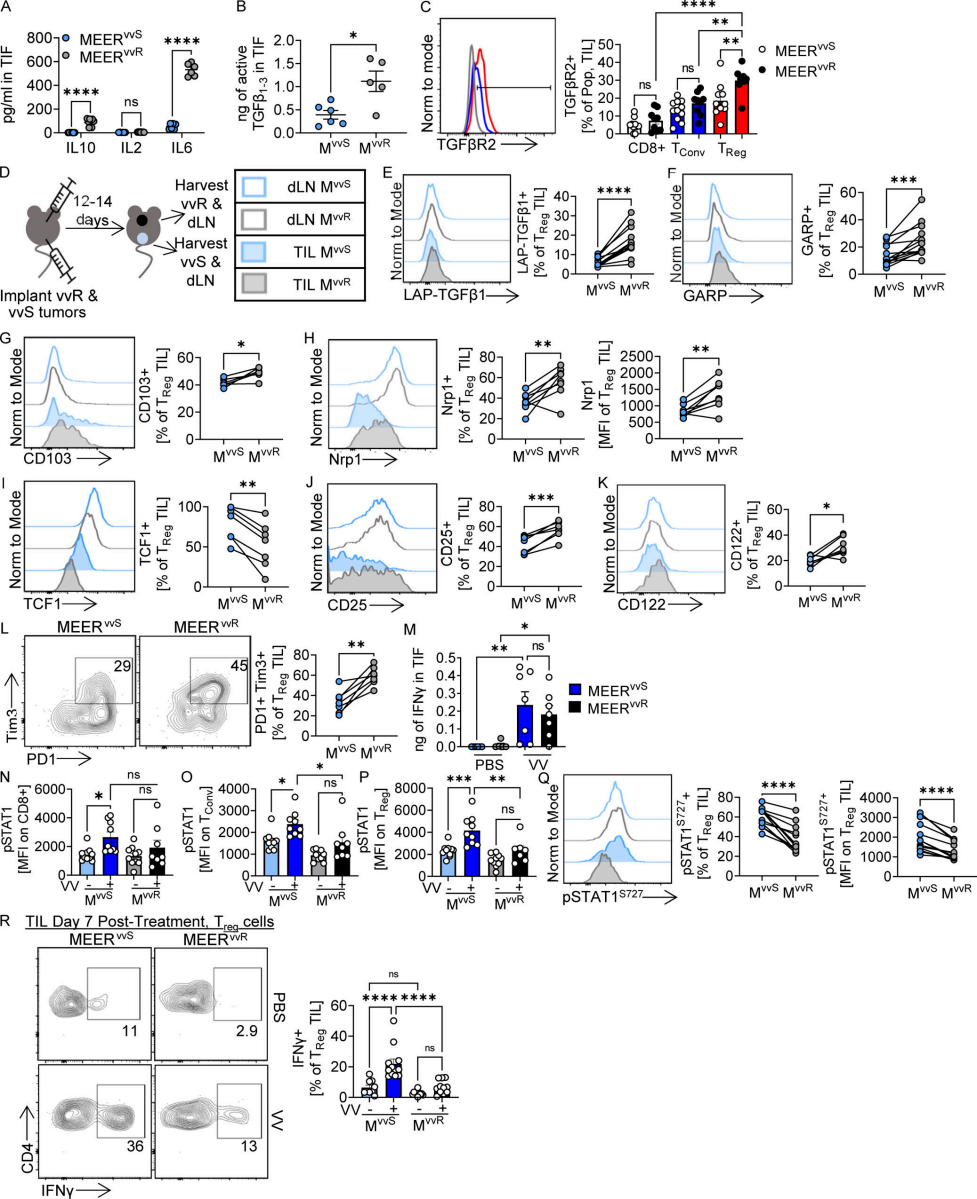
**T**_**reg**_ **cells in VV-resistant tumors have elevated TGFβ and a repressed response to IFNγ. (A)** Luminex cytokine analysis of TIF harvested from untreated MEER^vvR^ or MEER^vvS^ implanted in C57BL/6 mice. Three mice per group, three technical repeats per mouse. **(B)** Active TGFβ_1–3_ concentration in the TIF of untreated MEER^vvS^ or MEER^vvR^ tumors as determined by TGFβ reporter assay. **(C)** TGFβR2 expression on CD8^+^, Foxp3− T_conv_, or Foxp3+ T_reg_ cells in untreated MEER^vvR^ or MEER^vvS^ implanted in Foxp3-reporter mice. Representative histograms from a MEER^vvR^ tumor. 4 repeats, 10 M^vvS^, 9 M^vvR^ mice. **(D)** Experimental schema of E–L and Q. Repeated three times. **(E–L and Q)** Percentage of (E) LAP-TGFβ1+, (F) GARP+, (G) CD103+, (I) TCF1+, (J) CD25^+^, (K) CD122+, (L) PD-1+ Tim3+, and percentage and MFI of (H) Nrp1+ and (Q) pSTAT1+ T_reg_ cells by flow cytometry as in D. **(M)** IFNγ concentration in TIF of MEER^vvR^ and MEER^vvS^ at 7 d after treatment with PBS or VV as in Fig. 1. **(N–P)** Percentage of pSTAT1^S727^+ (N) CD8^+^ cells, (O) T_conv_ cells, and (P) T_reg_ cells in MEER^vvS^ or MEER^vvR^ tumors 7 d after treatment with VV as in Fig. 1. **(R)** Representative flow plots and percentage of IFNγ+ T_reg_ cells in MEER^vvS^ or MEER^vvR^ tumors 7 d after treatment with VV as in Fig. 1. Cells were restimulated with PMA/ionomycin direct ex vivo from tumors. M^vvS^ = MEER^vvS^, M^vvR^ = MEER^vvR^. Data represent two (A, B, and I–M), three (C, E–H, and Q), or four (N–P and R) independent experiments. Each point represents an individual mouse. *P < 0.05, **P < 0.01, ***P < 0.001, ****P < 0.0001 by two-way ANOVA with Sidak’s multiple comparison test (A), unpaired *T* test (B), one-way ANOVA with Sidak’s multiple comparison test paired *T* test (C, M–P, and R), or paired *t* test (E–L and Q). ns, non-significant. Error bars indicate SEMs.

**Figure S2.**
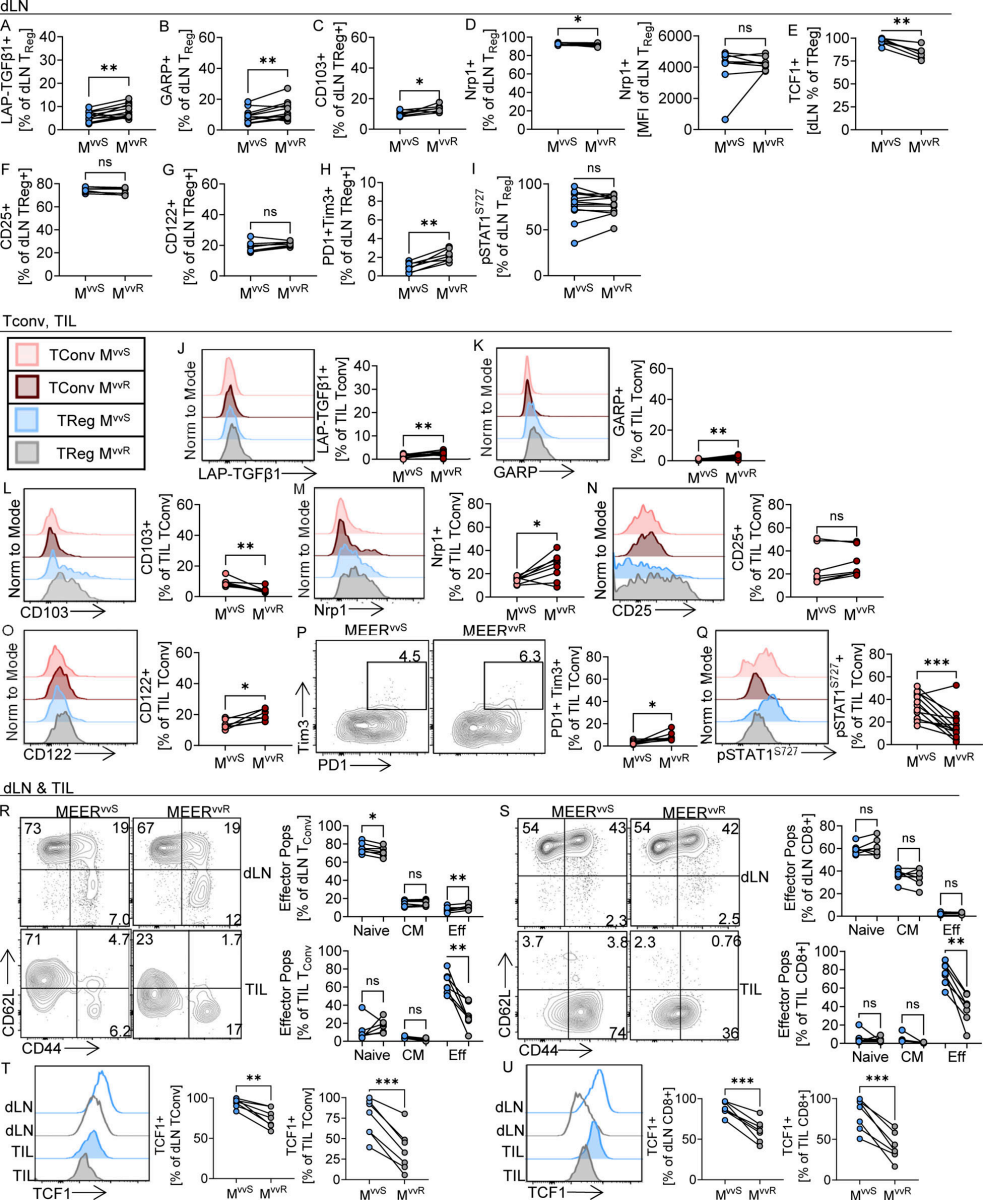
**Phenotyping observed in TIL T_reg_ cells is found in dLN and TIL T_conv_ to a lesser extent. (A–I)** Quantification of the percentages of dLN T_reg_ cells from paired tumors in Foxp3-Ametrine or Foxp3-RFP mice as in Fig. 2, D–N. (A) LAP-TGFβ1+, (B) GARP+, (C) CD103+, (D) Nrp1+, (E) TCF1+, (F) CD25^+^, (G) CD122+, (H) PD-1+ Tim3+, and (I) pSTAT1+. **(J–Q)** Representative flow plots and quantification of T_reg_ phenotyping markers on tumor-infiltrating T_conv_ cells as in Fig. 2, D–N. Representative histograms of tumor-infiltrating T_conv_ (Foxp3−) and T_reg_ (Foxp3+) cells are shown for comparison, and quantification axes are scaled for T_reg_ expression. Quantification and flow plots of (J) LAP-TGFβ1+, (K) GARP+, (L) CD103+, (M) Nrp1+, (N) CD25^+^, (O) CD122+, (P) Tim3, and PD-1 (Q) pSTAT1+ Tconv cells. **(R–U)** Representative flow plots and quantification in paired dLN and tumors as in Fig. 2, D–N of (R) CD62L and CD44 on T_conv_ cells, (S) CD62L and CD44 on CD8^+^ cells, (T) TCF1 on T_conv_ cells, and (U) TCF1 on CD8^+^ cells. CM, central memory CD62L+ CD44^+^; Eff, effector CD62L− CD44^+^; Naïve, CD62L+ CD44^−^. Data represent two (R–U) or three (A–Q) independent experiments. Each point represents an individual mouse *P < 0.05, **P < 0.01, ***P < 0.001 by paired *T* test (A–Q, T, and U) or one way ANOVA with Sidaks multiple comparisons test (R and S). ns, non-significant. Error bars indicate SEMs.

Having established that responses to OV in the MEER^vvS^ model were due to adaptive immunity, we performed cytokine analysis of the tumor interstitial fluid (TIF) to profile the balance of suppressive and stimulatory cytokines in the MEER^vvS^ and MEER^vvR^ tumors. In untreated tumors, we observed significantly higher levels of the protumorigenic IL-6 (Fisher et al., 2014; Fig. 2 A) and the suppressive cytokines IL-10 (Fig. 2 A) and TGFβ (Fig. 2 B) in MEER^vvR^ compared with MEER^vvS^. This corresponded with the RNA-seq data as both IL-6 and TGFβ can lead to changes in myc signaling and epithelial–mesenchymal transition in tumor cells (Massagué, 2008; Abaurrea et al., 2021). As IL-10 and TGFβ are associated with suppressive T_reg_ cells (Fu et al., 2004) and these cells expressed the highest levels of TGFβRII of the T cells in the TME (Fig. 2 C), we next investigated the phenotype of T_reg_ cells between the tumor types. To do so, we implanted both tumor types into contralateral sides of a single mouse to profile the effect of separate TME on the same immune system (Fig. 2 D). We observed that T_reg_ cells in MEER^vvR^ tumors had significantly higher surface latent associated protein (LAP)–TGFβ1 (Fig. 2 E) and the integrin GARP (Fig. 2 F), which is involved in the cleavage of LAP-TGFβ1 to the mature, active form of TGFβ1 (Wang et al., 2012). Both LAP-TGFβ1 and GARP are associated with more suppressive T_reg_ cells (Marie et al., 2005; de Streel et al., 2020). CD103, an integrin involved in cell–cell interactions, adhesion, and tissue homing, is maintained by TGFβ signaling in T_reg_ cells (Konkel et al., 2017). Expression of CD103 was also significantly higher in the MEER^vvR^ tumors (Fig. 2 G) and was greatly increased upon trafficking from the draining lymph node (dLN) to the tumor (Fig. 2 G and Fig. S2 C). T_reg_ cells in MEER^vvR^ also had a significantly higher surface expression of neuropilin-1 (Nrp1), which stabilizes T_reg_ cell function in cancer (Fig. 2 H; Delgoffe et al., 2013). Interestingly, TCF1 was significantly higher in T_reg_ cells in MEER^vvS^ tumors (Fig. 2 I). In T_reg_ cells, TCF1 deletion has been shown to upregulate Foxp3, IL2Ra (CD25), and TGFβ1, as well as other activation markers, and are superior at suppressing CD8 T cell proliferation and tumor control (Mammadli et al., 2023; Osman et al., 2021; Delacher et al., 2020). We find that in accordance with this, T_reg_ cells in MEER^vvR^ tumors have significantly higher CD25 and CD122 expression (Fig. 2, J and K) compared with T_reg_ cells in MEER^vvS^, as well as higher PD-1 and Tim3 expression (Fig. 2 L), markers of T_reg_ activation. Together these data suggest that T_reg_ cells in MEER^vvR^ are more suppressive and stable than in MEER^vvS^.

These trends hold true in the dLNs as well; however, the magnitude of expression is different (Fig. S2, A–H). While expression of TGFβ-dependent and activation markers are higher in the tumor (Fig. 2, E–K), Nrp1 and TCF1 are higher in the lymph node, as expected (Fig. S2, D and E). We also find that LAP-TGFβ1, GARP, Nrp1, CD122, and PD-1+Tim3+ expression are all higher on CD4^+^ Foxp3− conventional T cells (T_conv_) cells in MEER^vvR^ tumors compared with MEER^vvS^ (Fig. S2, J–P). However, this expression is on a much lower scale than what is observed in T_reg_ cells, so while it does appear that the suppressive TME affects T_conv_ cells in MEER^vvR^, it is not to the same degree as T_reg_ cells.

**Figure S3.**
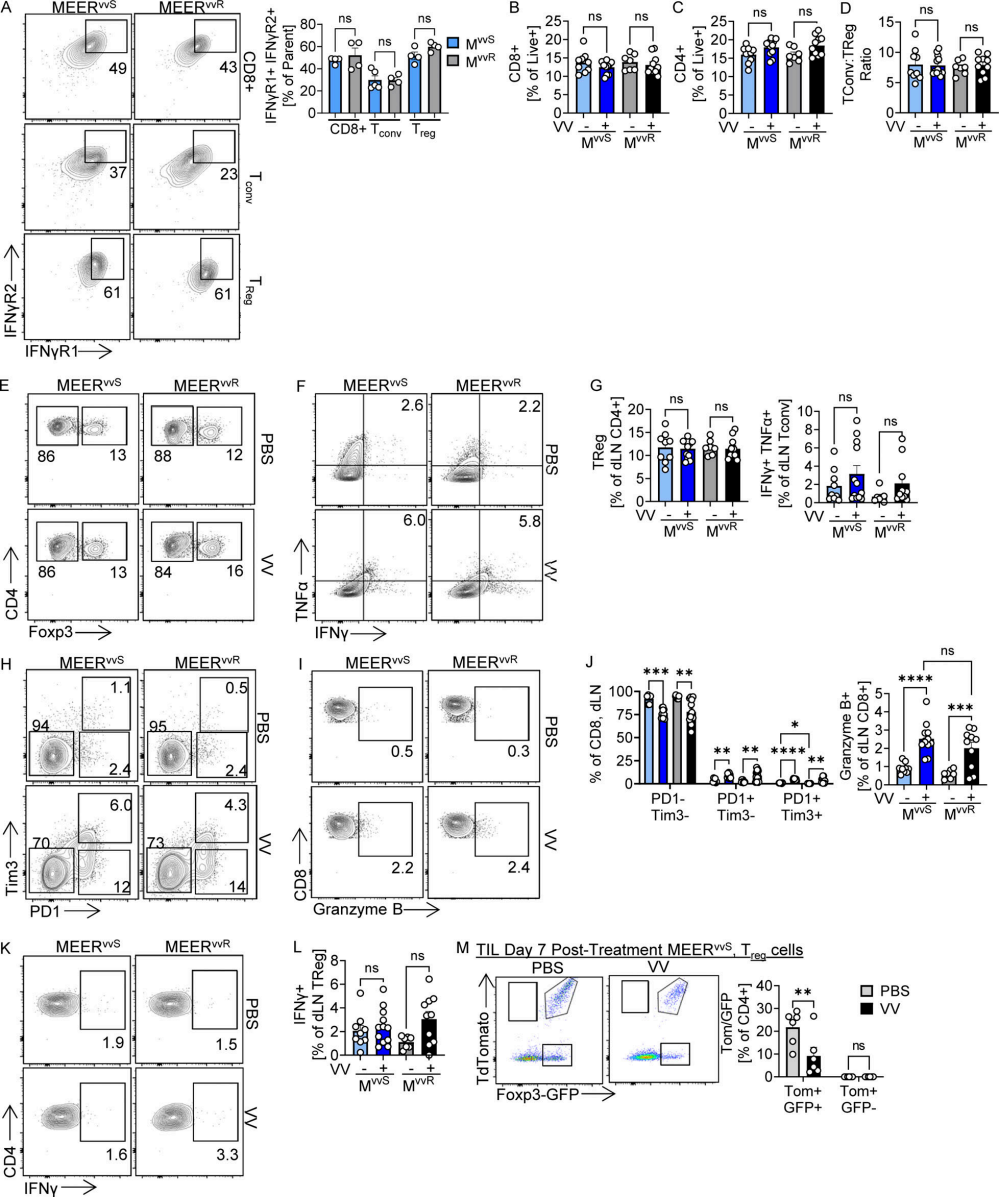
**T_conv_ and CD8 T cell phenotypic changes do not occur in the dLN. (A)** IFNγR1 and IFNγR2 expression in CD8^+^, T_conv_, or T_reg_ cells in untreated MEER^vvS^ or MEER^vvR^ implanted in Foxp3-Ametrine or Foxp3-RFP reporter mice. **(B–L)** Quantifications from dLN of unpaired treated tumors at 7 d after treatment as in Fig. 4. **(B)** CD8^+^ cells. **(C)** CD4^+^ cells. **(D)** T_reg_:T_conv_ ratio. **(E and F)** Representative flow plots of (E) Foxp3 expression in CD4^+^ cells and (F) TNFα and IFNγ in T_conv_ CD4^+^ cells. **(G)** Quantifications of E and F. **(H and I)** Representative flow plots of (H) PD-1 and Tim3 in CD8^+^ cells and (I) granzyme B in CD8^+^ cells. **(J)** Quantifications of H and I. **(K)** Representative flow plots of IFNγ in T_reg_ cells. **(L)** Quantification of K. **(M)** MEER^vvS^ tumors were implanted in *Foxp3*^*ERT2.GFP*^*Rosa26*^*LSL.TdTomato*^ mice. 2 d prior to VV treatment mice were given tamoxifen IP to induce TdTomato expression in T_reg_ cells. Tumors were treated with VV or PBS once an area of 20 mm^2^ was reached and 7 d after treatment were harvested for analysis. A representative flow plot and the quantified percentage of TdTomato and GFP expressing T_reg_ cells are shown. Data represent two (A and M) or six (B–K) independent experiments. Each point represents an individual mouse *P < 0.05, **P < 0.01, ***P < 0.001, ****P < 0.0001 by one-way ANOVA with Sidak’s multiple comparisons test (A–M). ns, non-significant. Error bars indicate SEMs.

We observed higher Nrp1 expression on T_reg_ cells in MEER^vvR^ tumors (Fig. 2 H) which acts to stabilize T_reg_ cells through its role as a co-receptor for TGFβ (Glinka and Prud’homme, 2008; Delgoffe et al., 2013). Nrp1, however, is also critical for maintaining T_reg_ cell stability in the presence of high IFNγ (Overacre-Delgoffe et al., 2017). T_reg_ cells that have low or no surface Nrp1 in the TME have increased IFNγ signaling and shift to a more Th1-like phenotype, producing IFNγ themselves and becoming less suppressive. As OVs, and poxviruses in particular, induce production of IFNγ within the TME (Worschech et al., 2009), we next isolated TIF from untreated and VV-treated tumors to determine IFNγ levels. Using an IFNγ ELISA, we found that 7 d after VV treatment there is significant induction of IFNγ in both sensitive and resistant tumors to similar levels (Fig. 2 M). Concomitantly, in MEER^vvS^ tumors, T_conv_, CD8^+^, and T_reg_ cells all have significant increases in STAT1 signaling 7 d after VV treatment compared with PBS, indicative of increased IFNγ response (Fig. 2, N–P). However, this was not observed in MEER^vvR^ tumors (Fig. 2, N–P), despite T cells within both tumor types expressing similar levels of the IFNγR (Fig. S3 A). Moreover, T_reg_ cells harbor elevated pSTAT1 even in the absence of VV treatment in MEER^vvS^ (Fig. 2 Q).

Consistent with low surface Nrp1 (Fig. 2 H) and high responsiveness to IFNγ (Fig. 2, P and Q), oncolytic VV treatment induced IFNγ production directly by the T_reg_ cells only within MEER^vvS^ tumors (Fig. 2 R), displaying a fragile T_reg_ cell phenotype in tumors with low TGFβ and high IFNγ. Thus, while both tumors harbor elevated concentrations of IFNγ after VV treatment, T cells in the resistant environment have decreased ability to respond to IFNγ, which helps T_reg_ cells avoid a fragile phenotype.

### T cell infiltrate in MEER^vvR^ tumors has reduced functionality compared with MEER^vvS^

As the T_reg_ cells in MEER^vvR^ appear to be more suppressive and stable, we next investigated other T cell types (effector CD8^+^ and T_conv_ populations) in the two models. We found that in MEER^vvR^ tumors, there was a significant reduction in effector T cells (CD62L− CD44^+^, Fig. S2, R and S). Interestingly, there is also a small but significant decrease in naïve (CD62L+ CD44^−^) and increase in effector T_conv_ cells in the dLN of MEER^vvR^ (Fig. S2 R). We also found a decrease in both dLN and tumor of TCF1 expression in T_conv_ (Fig. S2 T) and CD8^+^ (Fig. S2 U). TCF1 is a marker of stemness and is important for differentiation of T_conv_ cells and maintenance of a stem-like CD8^+^ T cell population in the tumor, which is critical for response to checkpoint blockade (Zhao et al., 2022). Together, these data show that, concordant with increased T_reg_ cell suppression, there is a reduced effector response in the tumor of the MEER^vvR^.

**Figure 3.**
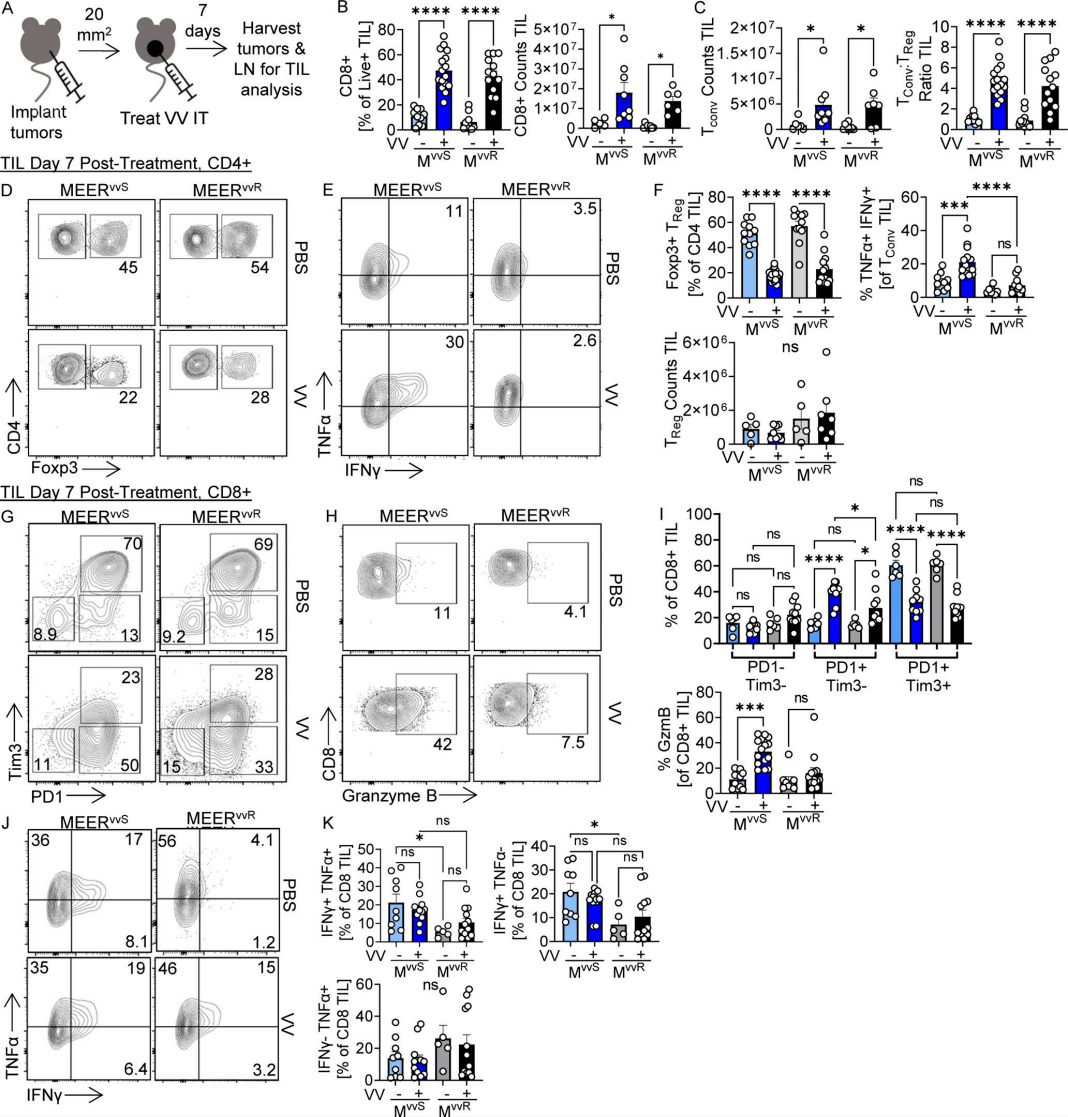
**Effector T cells infiltrating VV-treated MEER**^**vvS**^ **tumors are more functional than those infiltrating non-responsive tumors. (A)** Experimental schema for B–I. Foxp3-Ametrine or Foxp3-RFP reporter mice implanted intradermally with MEER^vvS^ or MEER^vvR^ were treated with an IT injection of VV at 2.5 × 10^5^ PFU/mouse or PBS control. Tumors and dLNs were harvested 7 d after treatment for phenotypic analysis. **(B and C)** (B) Percentage and total counts of CD8^+^ T cells and the (C) counts and ratio of T_conv_ cells to T_reg_ cells in treated tumors. **(D)** Representative flow plots of CD4^+^ Foxp3− T_conv_ and Foxp3+ T_reg_ cells. **(E)** Representative flow plots of TNFα and IFNγ production in T_conv_ cells after direct ex vivo restimulation with PMA and ionomycin. **(F)** Quantifications of D and E. **(G)** Representative flow plots of PD-1 and Tim3 expression on CD8^+^ cells. **(H)** Representative flow plots of granzyme B production in CD8^+^ T cells after direct ex vivo restimulation with PMA and ionomycin. **(I)** Quantifications of G and H. **(J)** Representative flow plots of TNFα and IFNγ production in CD8^+^ cells after direct ex vivo restimulation with PMA and ionomycin. **(K)** Quantification of J. Data represent six independent experiments (B–F and H–K) or four independent experiments (G). Each point represents an individual mouse. *P < 0.05, ***P < 0.001, ****P < 0.0001 by one-way ANOVA with Sidak’s multiple comparison test (B, C, F, I, and K). ns, non-significant. Error bars indicate SEMs.

To determine the effect of T_reg_ cell stability after VV treatment, we profiled the immune infiltrate 7 d after VV treatment (Fig. 3 A), when tumors were still of comparable size. Regardless of tumor type, we observed an increase in both absolute numbers and the percentage of CD8^+^ T cells (Fig. 3 B). We also observed an increased in the counts of T_conv_ cells and the ratio of T_conv_:T_reg_ cells in VV-treated tumors (Fig. 3 C). No significant change was observed in counts of T_reg_ cells, while percentage was decreased (Fig. 3, D and F).

While effector T cell influx occurred in all tumors, the functionality of these cells was markedly different. VV-treated MEER^vvS^ tumors harbored more polyfunctional T_conv_ cells (Fig. 3, E and F). After VV treatment, there was a significant increase in PD-1+Tim3− CD8^+^ T cells and a significant decrease in PD-1+Tim3+ exhausted CD8^+^ T cells (Fig. 3, G and I) in both tumors; however, only in MEER^vvS^ was there an increase in granzyme B+ producing CD8^+^ T cells (Fig. 3, H and I) compared with MEER^vvR^. Thus, tumors fated to experience a CR to VV have functional differences within the tumor infiltrate. There were no significant differences between MEER^vvS^ and MEER^vvR^ in infiltration of T cells or functionality in the dLN, showing that these functionality differences are maintained in the tumor (Fig. S3, B–L). Interestingly, while there was no increase in cytokine production by CD8^+^ T cells after VV treatment (Fig. 3, J and K), T cells in MEER^vvS^ produced significantly more IFNγ in the PBS condition than MEER^vvR^. This further suggests that CD8^+^ T cells in sensitive tumors are more functional, even at baseline.

As we saw decreases in the T_reg_ cell population after treatment (Fig. 3 F), we wanted to address the possibility that VV treatment may cause loss of Foxp3 and generate “ex-T_reg_” cells. To do so, we used a *Foxp3*^*Cre.ERT2.GFP*^ × *Rosa26*^*LSL.Td.Tomato*^ mouse model to verify that these cells were not losing Foxp3 expression (Fig. S3 M). These mice were treated with tamoxifen 2 d prior to VV treatment to activate Cre recombinase and induce irreversible TdTomato signal in addition to their Foxp3-GFP reporter. If a cell was expressing Foxp3 before treatment and lost it, becoming an ex-T_reg_, it would continue to express TdTomato but lose GFP expression. 7 d after treatment, we observed no TdTomato+ GFP− cells in these mice, suggesting that these cells do not lose Foxp3 expression. The GFP+ Tomato− cells observed are newly generated T_reg_ cells that entered the tumor after the tamoxifen treatment.

Together, our data suggest the MEER^vvS^ TME harbors T_reg_ cells primed to be inflammatory, such that IFNγ induced by viral infection promotes a state of T_reg_ cell fragility, resulting in a less immunosuppressive environment and greater effector function of the newly induced T cell infiltrate.

### High intratumoral TGFβ reduces sensitivity to IFNγ

**Figure 4.**
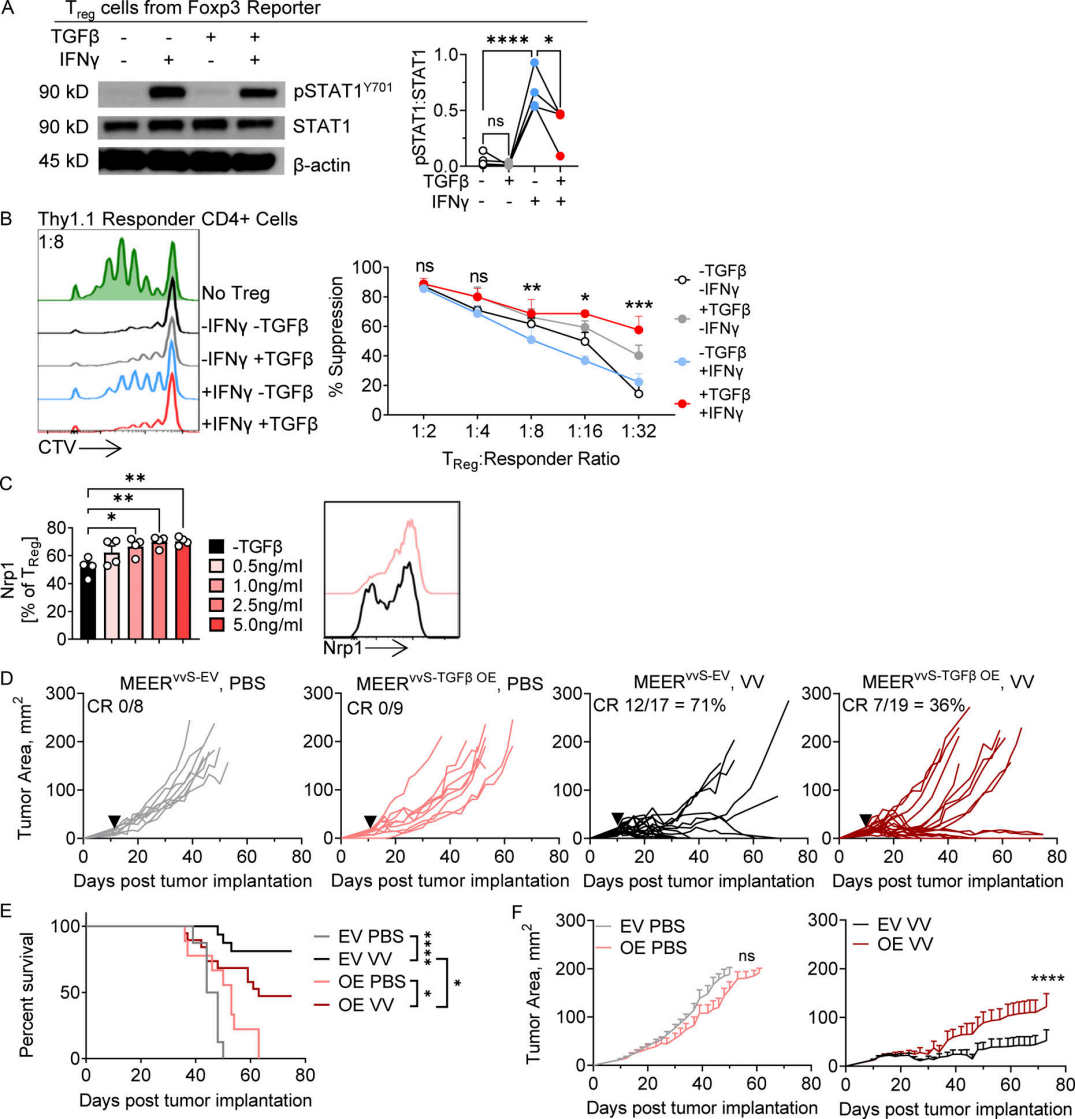
**TGFβ limits IFNγ signaling and increases T**_**reg**_ **cell stability. (A)** Immunoblot and densitometry of pSTAT1^Y701^, STAT1, and β-actin in T_reg_ cells sorted from spleen and lymph node of a Foxp3 reporter mouse and treated for 30 min with IFNγ, TGFβ1, or both. **(B)** Quantification and Cell Trace Violet (CTV) plots of the proliferation of stimulated Thy1.1+ CD4 responder cells in the presence of suppressing T_reg_ cells at the 1:8 T_reg_ cell:responder ratio in an in vitro suppression assay. Percent suppression is normalized to the proliferation index of stimulated CD4^+^ responder control without T_reg_ cells. T_reg_ cells were sorted from spleen and lymph node of a Foxp3-reporter mouse and then cultured for 3 d in IFNγ, TGFβ, or both. Cells were then sorted again to purify Foxp3+ T_reg_ cells and then co-cultured in the suppression assay with CTV-labeled responder CD4^+^ cells. **(C)** Surface Nrp1 expression on sorted T_reg_ cells from spleen and lymph node of a Foxp3-reporter mouse cultured in vitro in varying TGFβ concentrations for 48 h **(D–F)** An EV control and TGFβ_1_ overexpressing (TGFβ OE) line were generated from the MEER^vvS^ line. **(D)** Tumor growth of C57BL/6 mice implanted intradermally with MEER^vvS-EV^ or MEER^vvS-TGFβ OE^ and, when tumors were ∼20 mm^2^, treated with a single IT injection of VV at 2.5 × 10^5^ PFU/mouse or PBS control (black arrowhead). Mice were sacrificed when tumors reached 15 mm in any direction. **(E)** Survival of D. **(F)** Average tumor growth of MEER^vvS-EV^ and MEER^vvS-TGFβ OE^ as in D. Data represent two (C), four (A and D–F), or five (B) independent experiments; each point or line represents an individual mouse (A–D). *P <0.05, **P < 0.01, ***P < 0.001, ****P < 0.0001 by one-way ANOVA with Sidak’s multiple comparison test (A and C), paired *T* test (B), Mantel-Cox test (E), or mixed effects analysis (F). ns, non-significant. Error bars indicate SEMs. Source data are available for this figure: SourceData F4.

We next asked if high intratumoral TGFβ in MEER^vvR^ tumors was the cause of the reduced responsiveness of immune cells to inflammatory signals after VV treatment. TGFβ is a pleiotropic cytokine with very immunosuppressive effects within the TME (Massagué, 2008). TGFβ can both directly suppress T cell infiltration and activity and differentiate and stabilize T_reg_ cells. Further, consistent with previous reports in conventional T cells, TGFβ treatment of T_reg_ cells directly inhibits STAT1 signaling upon IFNγ treatment (Fig. 4 A). This has been shown to occur through induction of the protein tyrosine phosphatase SHP-1, which reduces JAK-STAT signal (Reardon and McKay, 2007; Park et al., 2005). It has been previously shown that IFNγ can reduce the suppressive capacity of T_reg_ cells (Overacre-Delgoffe et al., 2017). Culturing T_reg_ cells in the presence of TGFβ and IFNγ for 2 d prior to use in an in vitro suppression assay fully restores their suppressive capacity compared with IFNγ culture alone (Fig. 4 B). This shows that TGFβ alone is enough to interrupt IFNγ signaling and maintain T_reg_ cell function. As Nrp1 has previously been shown to enhance T_reg_ cell stability in the TME, we tested if TGFβ alone was sufficient to induce increased surface Nrp1 on T_reg_ cells. Indeed, TGFβ treatment of T_reg_ cells in vitro leads to a significant increase in surface Nrp1 (Fig. 4 C). Altogether, TGFβ may be playing a dominant role in establishing an environment resistant to OV therapy by stabilizing T_reg_ cells and inhibiting sensing of virus-induced IFNγ on all T cells.

**Figure S4.**
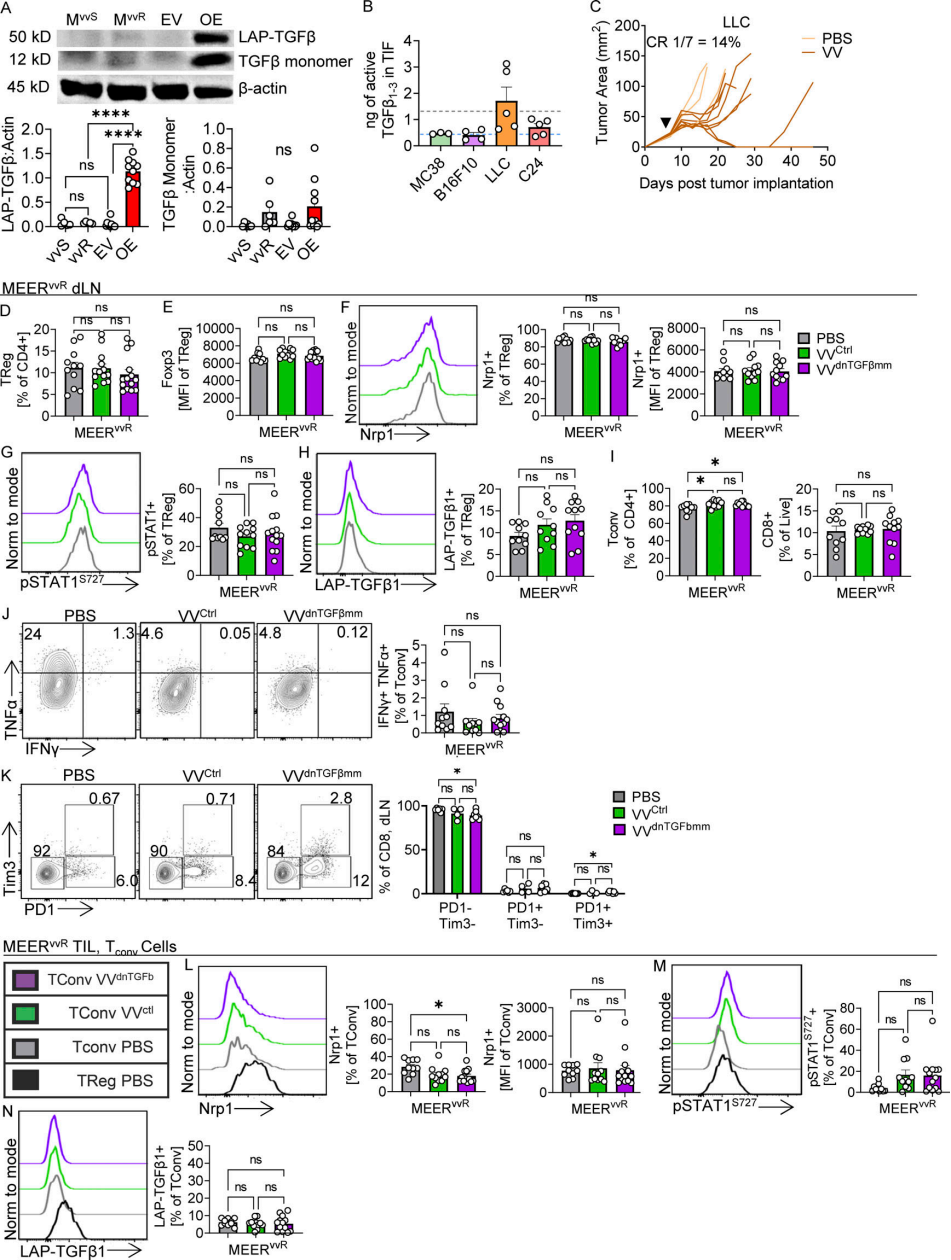
**VVdnTGFbmm only affects T**_**reg**_** cell phenotype in the tumor. (A)** Western blot for TGFβ in EV control and TGFβ_1_ overexpressing (TGFβ OE) MEER^vvS^ lines and MEER^vvR^ and MEER^vvS^ as in Fig. 3. **(B)** Active TGFβ_1–3_ levels measured in TIF of LLC, MC38 (colon adenocarcinoma), B16-F10 (melanoma), and C24 (*Pten*^*–/–*^*Braf*^*V600E*^ melanoma) tumors in C57Bl/6 mice as in Fig. 2 B. The average TGFβ concentration of MEER^vvS^ (light blue) and MEER^vvR^ (gray) from Fig. 2 B are overlaid as dotted lines. **(C)** Growth curve of LLC tumors treated with PBS or VV (black arrowhead) as in Fig. 1. **(D–N)** Representative flow plots and quantification of dLN (D–K) and T_reg_ phenotyping markers on tumor infiltrating T_conv_ cells (L–N) in Foxp3-Ametrine or Foxp3-RFP mice as in Fig. 7. Quantification of dLN (D) percent Foxp3+ of CD4^+^ and (E) MFI of Foxp3. Quantification and representative flow plots of (F) Nrp1+, (G) pSTAT1+, and (H) LAP-TGFβ1 on dLN T_reg_ cells. Quantification of dLN (I) percent Foxp3− of CD4^+^ and CD8^+^. Quantification and representative flow plots of (J) TNFα and IFNγ in T_conv_ cells with direct ex vivo PMA/ionomycin stimulation and (K) PD-1 and Tim3 on CD8^+^ cells in dLN. Quantification and representative flow plots of (L) Nrp1+, (M) pSTAT1+, and (N) LAP-TGFβ1 on TIL T_conv_ cells. Data represent two (A–C) or four (D–N) independent experiments. Each dot or line represents a technical repeat (A) or mouse (B–N). *P < 0.05 by one-way ANOVA with Sidak’s multiple comparisons test. ns, non-significant. Source data are available for this figure: SourceData FS4.

To test the effects of TGFβ directly on responsiveness to VV treatment, we overexpressed TGFβ1 into the MEER^vvS^ tumor line to generate MEER^vvS-TGFβ OE^ and an EV control (MEER^vvS-EV^, Fig. S4 A). These tumors were treated with an IT injection of PBS or VV as described previously, and we observed that while the MEER^vvS-EV^ maintained a CR rate of ∼70% as previously reported (Fig. 1 A), the MEER^vvS-TGFβ OE^ tumors had only 36% of tumors undergo a CR (Fig. 4 D). The VV-treated TGFβ1 overexpressing tumors had a significantly reduced survival (Fig. 4 E) as well as an increased tumor growth (Fig. 4 F) compared with VV-treated EV tumors. Thus, elevating TGFβ within the TME of the sensitive MEER tumor was sufficient to reduce the responsiveness to VV therapy.

**Figure S5.**
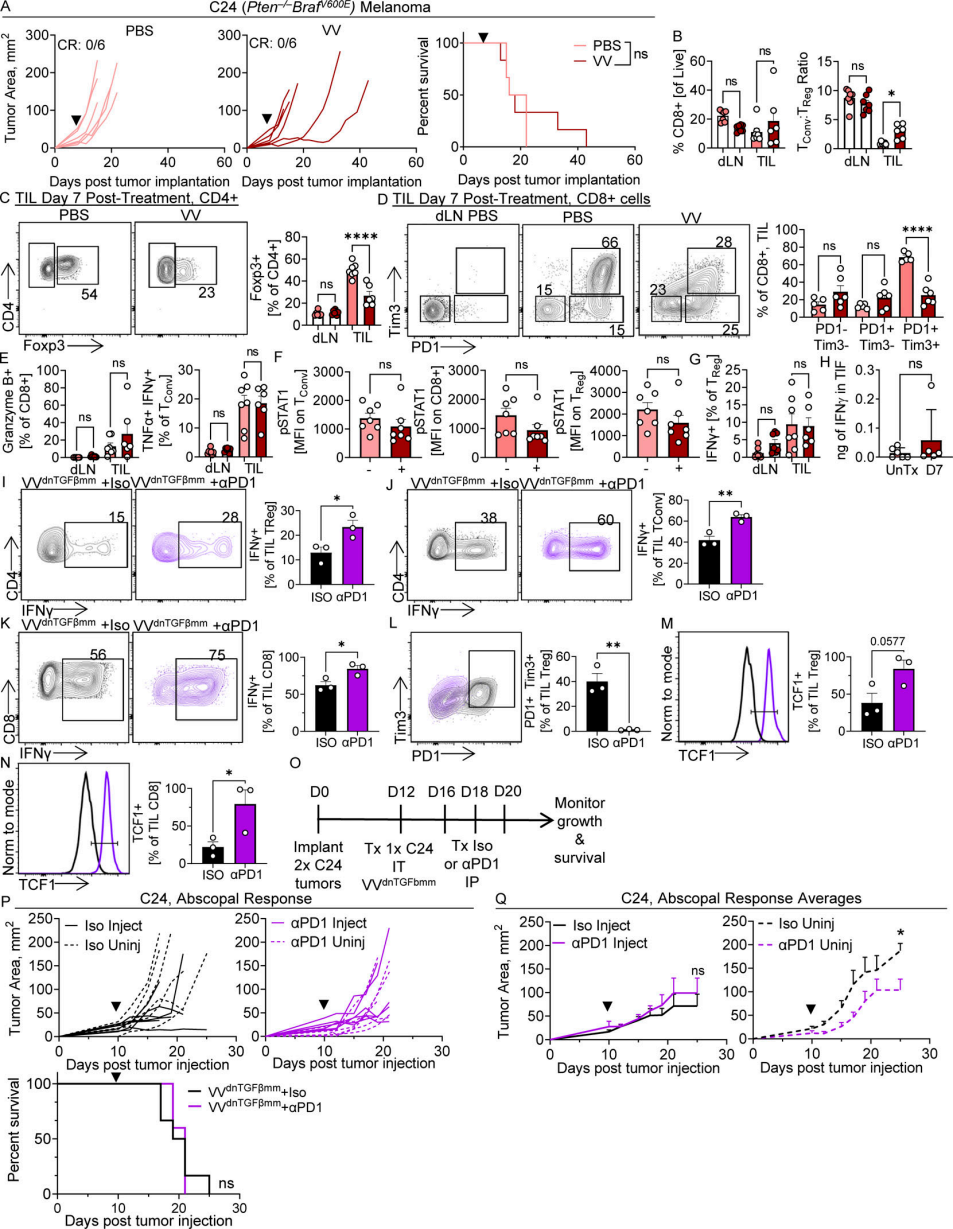
**C24 melanoma model has a similar phenotype to MEER^vvR^ after VV treatment. (A)** Tumor growth (left, middle) and survival (right) of C57BL/6 mice implanted intradermally with C24 and, when tumors were ∼20 mm^2^, treated with a single IT injection of VV at 2.5 × 10^5^ PFU/mouse or PBS control (black arrowhead). Mice were sacrificed when tumors reached 15 mm in any direction. **(B)** Foxp3-Ametrine or Fpxp3-RFP reporter mice implanted intradermally with C24 were as in A. Tumors and lymph nodes were harvested 7 d after treatment for phenotypic analysis. Percentage and total counts of CD8^+^ T cells and the ratio of T_conv_ cells to T_reg_ cells in treated tumors. **(C)** Percentage of Foxp3+ CD4^+^ T_conv_ cells. **(D)** PD-1 and Tim3 expression on CD8^+^ cells. **(E and F)** (E) Production of granzyme B in CD8^+^ T cells and (F) MFI of pSTAT1 on T_conv_, CD8^+^, and T_reg_ cells as in B. **(G)** Production of IFNγ in T_reg_ cells as in B after restimulation with PMA and ionomycin as in B. **(H)** IFNγ measured by ELISA from the TIL of CL24 untreated and 7 d after VV treatment as in B. Mice were implanted with C24 and treated with VV^dnTGFβmm^ and αPD-1 as in Fig. 7. At day 8 after VV treatment, after three doses of αPD-1 were received, tumors were harvested for TIL analysis. **(I–N)** Representative flow plots and quantifications of (I) IFNγ+ T_reg_ cells, (J) IFNγ + T_conv_ cells, (K) IFNγ + CD8^+^ cells, (L) Tim3+ PD-1+ T_reg_ cells, (M) TCF1+ T_reg_ cells, and (N) TCF1+ CD8^+^ cells. Cytokine analysis was performed direct ex vivo with PMA and ionomycin restimulation. **(O)** Schematic for P and Q. **(P)**Tumor growth and survival (bottom) of C57BL/6 mice implanted intradermally with bilateral C24 and, when tumors were ∼20 mm^2^, one was treated with a single IT injection of VV^dnTGFbmm^ (injected) at 2.5 × 10^6^ PFU/mouse (black arrowhead). Mice were sacrificed when either tumor reached 15 mm in any direction. Starting at 4 d after VV or PBS treatment, mice were given anti-PD-1 or isotype control IP three times a week. **(Q)** Average growth of H. Data represent two independent experiments. Each point or line represents an individual mouse (A–P). *P < 0.05, **P < 0.01, ****P < 0.0001 by Welch’s *T* test (B–H), Mantel-Cox test (A and P), unpaired *t* Test (I–N), or mixed-effects analysis (Q). ns, non-significant. Error bars indicate SEMs.

We also tested the levels of active TGFβ_1-3_ in other tumor models that are both sensitive and resistant to VV therapy to determine if this was a common resistance mechanism. We found that in MC38 (Rivadeneira et al., 2019) and B16-F10 (Deng et al., 2019; Jeon et al., 2022; Kim et al., 2009), which are sensitive and partially responsive to VV therapy, respectively, there are low levels of TGFβ, similar to what is found in MEER^vvS^ (Fig. S4 B). Lewis lung adenocarcinoma (LLC; Fig. S4 C) and clone24 (C24; Fig. S5 A, Rivadeneira et al., 2019), which are resistant to VV therapy, contain higher concentrations of TGFβ than MEER^vvS^, similar to and exceeding MEER^vvR^ (Fig. S4 B). This suggests that at least in the models tested, the level of TGFβ_1-3_ in the tumor may contribute to resistance to oncolytic VV therapy.

### An oncolytic vaccinia that produces a TGFβRII inhibitor renders MEER^vvR^ sensitive to treatment

**Figure 5.**
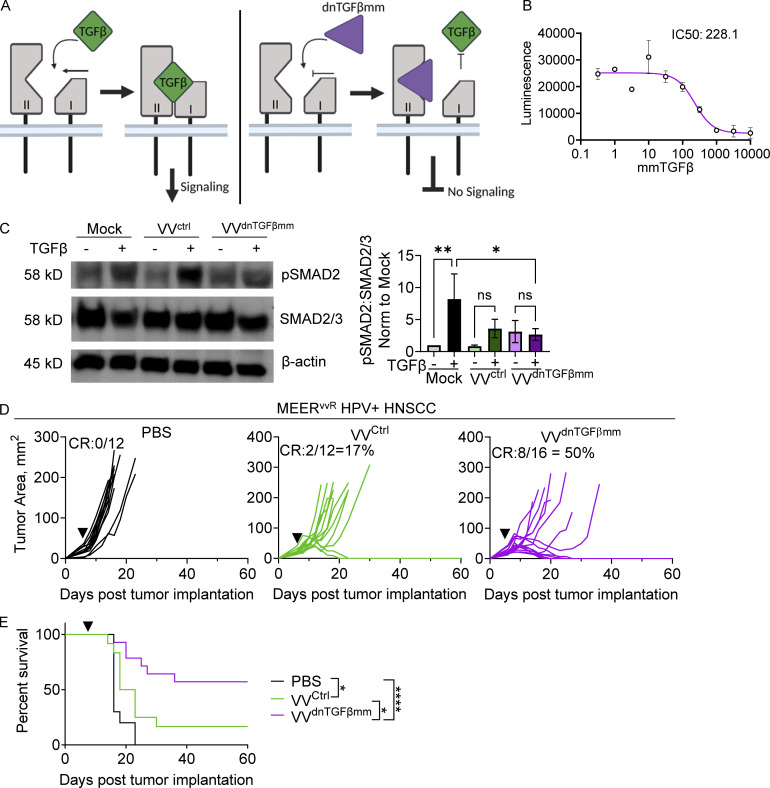
**Engineering a genetically encoded TGFβ signaling inhibitor (dnTGFβmm) into VV enhances response. (A)** Schematic of the mode of dnTGFβmm inhibition. **(B)** Luminescence of stably transfected TGFβ reporter HEK293 cells treated with increasing doses of recombinant dnTGFβmm and stimulated with 10 pM TGFβ3. The data was fit to standard models for ligand inhibitory activity (IC50). **(C)** Immunoblot and densitometry of pSmad2 signaling, downstream of TGFβ, in T cells isolated from spleen and lymph node of wild-type mice, treated with recombinant TGFβ1 and supernatant from HeLa cells infected with VV^ctrl^, VV^dnTGFβmm^, or mock-infected. **(D and E)** Tumor growth (D) and survival (E) of MEER^vvR^-bearing C57BL/6 mice treated with an IT injection of PBS, VV^ctrl^, or VV^dnTGFβmm^ at 2.5 × 10^6^ PFU/mouse (black arrowhead). Data represent three independent experiments with three technical replicates (B) or individual mice (C and D). In D, each line represents an individual mouse. *P < 0.05, **P < 0.01, ****P < 0.0001 by one-way ANOVA (C) or Mantel-Cox test (E). ns, non-significant. Error bars indicate SEMs. Source data are available for this figure: SourceData F5.

We next asked whether TGFβ could be targeted to overcome OV resistance. TGFβ has been difficult to target successfully in the clinic as systemically administered agents must balance potency with toxicity. Indeed, systemic TGFβ targeting can induce autoimmune side effects and cardiac toxicity (Teixeira et al., 2020). However, as OVs can be used to deliver genetic payload, we reasoned a genetically encoded TGFβ inhibitor would be restricted to the TME and thus could be exceptionally potent. We engineered VV to express a dominant negative, mini-monomeric TGFβ (termed dnTGFβ^mm^) within the TK locus. This dnTGFβ^mm^ was derived from the original variant of a mutant form of TGFβ containing structurally guided mutations to prevent dimerization, generated by Kim et al. (2017). This small monomeric TGFβ fragment binds to TGFβRII, preventing the recruitment of TGFβRI, thus inhibiting receptor activity, and outcompeting endogenous TGFβ_1-3_ for receptor binding (Fig. 5 A). Treatment with this virus would force tumor cells to produce this potent TGFβ inhibitor within the local TME.

We confirmed inhibitory activity of recombinant dnTGFβ^mm^ using both TGFβ reporter cell lines (Fig. 5 B) and supernatants harvested from VV^dnTGFβmm^, VV^ctrl^, or mock-infected HeLa cells to confirm a reduction in TGFβ signaling within T cells (Fig. 5 C). TGFβRII expression on the surface of T cells in the MEER^vvR^ tumors was most highly expressed by T_reg_ cells but was evident on all tumor-infiltrating T cells (Fig. 2 C).

We then asked whether resistant tumors could be rendered sensitive to VV if TGFβ inhibition was encoded in the virus. Strikingly, elite responses were regained in MEER^vvR^ upon treatment with VV^dnTGFβmm^, resulting in over 50% long-term CRs (Fig. 5 D) as well as a significant increase in survival (Fig. 5 E) compared with VV^ctrl^. Importantly, these mice had no autoimmune or other toxicity-induced side effects.

### VV^TGFβmm^ increases T_reg_ cell fragility in MEER^vvR^ tumors

To understand if there were phenotypic changes to the T_reg_ cells after VV^dnTGFβmm^ treatment, we analyzed the tumor infiltrate at 4 and 7 d after treatment in MEER^vvR^ tumors. The 4-d timepoint was chosen as this is during the peak of viral infection and as such the inhibitor should still be produced and present in the TME, while at the 7-d timepoint, we examined the phenotype of the new T cell infiltrate.

**Figure 6.**
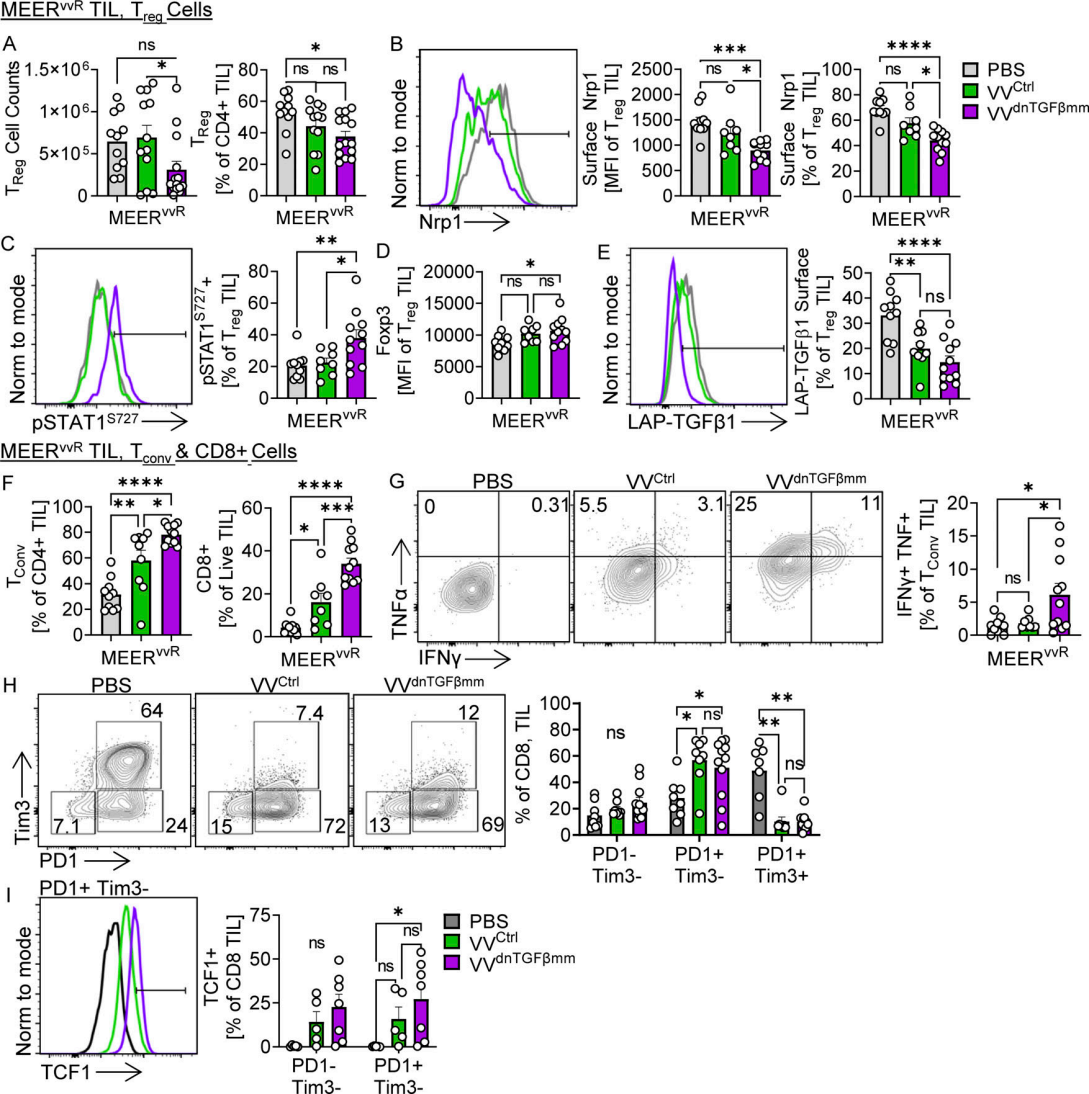
**Viral delivery of TGFβ inhibition alleviates immunosuppressive T**_**reg**_ **cells in resistant tumors.** Foxp3-Ametrine or Foxp3-RFP mice implanted intradermally with MEER^vvS^ or MEER^vvR^ were treated with an IT injection of VV^ctrl^ or VV^dnTGFβmm^ at 2.5 × 10^6^ PFU/mouse or PBS control. **(A–E)** Tumors and lymph nodes (Fig. S5) were harvested 4 (A) or 7 (B–E) d after treatment for phenotypic analysis. **(A)** Percentage and total counts of T_reg_ cells at day 4 after treatment. **(B)** Percentage and MFI of Nrp1+ T_reg_ cells at day 7. **(C)** Percentage of pSTAT1^Ser727^+ T_reg_ cells at day 7. **(D)** MFI of Foxp3 in T_reg_ cells at day 7. **(E)** Percentage of LAP-TGFβ1+ T_reg_ cells at day 7. **(F)** Percentage of T_conv_ cells and CD8^+^ cells 7 d after treatment. **(G)** Production of TNFα and IFNγ in T_conv_ cells from treated tumors after restimulation with PMA and ionomycin. **(H)** Percentage of PD-1- and Tim3-expressing CD8^+^ cells 7 d after treatment. **(I)** Percentage of TCF1+ in PD-1 and Tim3 CD8^+^ populations 7 d after treatment, representative plot of PD-1+Tim3− cells. Data represent three (H and I) or four (A–G) independent experiments. Each point represents an individual mouse. *P < 0.05, **P < 0.01, ***P < 0.001, ****P < 0.0001 by one-way ANOVA (A–G) or two-way ANOVA (H and I) with Tukey’s multiple comparison test. ns, non-significant. Error bars indicate SEMs.

4 d after treatment, we found a significant decrease in T_reg_ cells in the VV^dnTGFβmm^ group by counts compared with VV^ctrl^ (Fig. 6 A); however, we did not observe changes in T_reg_ cell phenotype. 7 d after treatment, we found T_reg_ cells in tumors treated with VV^dnTGFβmm^ had significantly lower surface Nrp1 by percentage and human papilloma virus (MFI) than PBS or VV^ctrl^-treated tumors (Fig. 6 B). This was consistent with our previous data showing that Nrp1 is increased on the surface of T_reg_ cells when cultured with TGFβ. Consistent with the notion that TGFβ inhibits IFNγ signaling in T_reg_ cells, these T_reg_ cells had significantly higher STAT1 signaling than T_reg_ cells in VV^ctrl^-treated tumors (Fig. 6 C) while retaining similar Foxp3 expression to T_reg_ cells in VV^ctrl^ tumors (Fig. 6 D). Increased IFNγ signaling while maintaining Foxp3 expression suggests these T_reg_ cells become fragile once TGFβ signaling is lost. T_reg_ cells in VV^dnTGFβmm^-treated tumors also had significantly lower surface LAP-TGFβ1, suggesting these cells may be less suppressive (Fig. 6 E). Together these data show that reducing TGFβ signaling in T_reg_ cells sensitizes them to the increased IFNγ after VV treatment, causing T_reg_ fragility.

Also 7 d after treatment, we found a significant increase in the percentage of CD4^+^ T_conv_ cells and CD8^+^ cells in the VV^dnTGFβmm^ group compared with both VV^ctrl^ and PBS (Fig. 6 F). Commensurate with induction of T_reg_ fragility, T_conv_ cells produced significantly increased levels of TNFα and IFNγ (Fig. 6 G). While treatment with both VV^ctrl^ and VV^dnTGFβmm^ lead to increases in PD-1+Tim3− CD8^+^ T cells and reduced PD-1+Tim3+ exhausted T cells (Fig. 6 H), only VV^dnTGFβmm^ increased TCF1 expression (Fig. 6 I). This phenocopies what was observed in VV-treated MEER^vvS^ tumors (Fig. 3). These changes were again limited to the tumor, where the inhibitor is found (Fig. S4, D–K). Interestingly, Nrp1 was also significantly decreased on T_conv_ cells in the tumor (Fig. S4 L), showing that reduced TGFβ signaling may affect other cell types as well, although overall Nrp1 levels were overall much lower than what is observed on T_reg_ cells.

Targeting TGFβ can increase T_reg_ sensitivity to IFNγ, leading to a less suppressive T_reg_ phenotype, ultimately resulting in an environment more responsive to immunomodulation.

### VV^dnTGFβmm^ can synergize with αPD-1

We also repeated these experiments in the C24 melanoma model to determine if this phenotype occurred in other VV-resistant tumors. This model is a single-cell derivative from a melanoma forming in the *Pten*^f/f^*Braf*^LSL^^-V600E^*Tyr*^Cre.ER^ mouse model and is resistant to most therapies, including PD-1 blockade, T cell therapies, and OVs (Najjar et al., 2019; Rivadeneira et al., 2019). Consistent with our previous work, VV induced tumor growth inhibition but did not result in any CRs (Fig. S5 A; Rivadeneira et al., 2019). We observed a similar T cell phenotype to the resistant MEER^vvR^ model, wherein after treatment we observe modest increases in CD8^+^ T cells and increased T_conv_:T_reg_ ratio (Fig. S5, B and C) but no difference in functionality of those cells (Fig. S5, E and F). We observe similar TGFβ levels to MEER^vvR^ (Fig. S4 B), as well as no increase in IFNγR activity in T cells (Fig. S5 F) or IFNγ production by T_reg_ cells (Fig. S5 G). However, we do not observe a statistically significant increase in TIF IFNγ after VV treatment in the C24 (Fig. S5 H).

**Figure 7.**
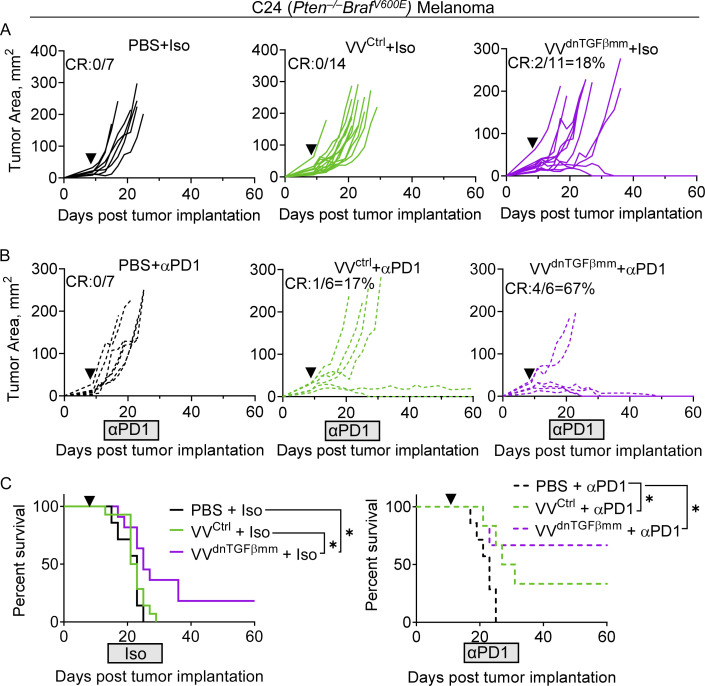
**VV**^**dnTGFβmm**^ **synergizes with anti-PD-1 in an immunotherapy resistant melanoma model. (A)** Tumor growth of C57BL/6 mice implanted intradermally with C24 and, when tumors were ∼20 mm^2^, treated with a single IT injection of VV^ctrl^, VV^dnTGFbmm^ at 2.5 × 10^6^ PFU/mouse, or PBS control (black arrowhead). Mice were sacrificed when tumors reached 15 mm in any direction. **(B)** Tumor growth of mice treated with virus as in A. Starting at 4 d after VV or PBS treatment, mice were given anti-PD-1 or isotype control IP three times a week for a total of seven treatments (gray box). **(C)** Survival as in A and B. Data represent three independent experiments (A–C). Each line represents an individual mouse (A and B). *P < 0.05 by Mantel-Cox test (C). Error bars indicate SEMs.

We next treated C24 with VV^dnTGFβmm^. In this model, we observed ∼20% CRs and a significant increase in survival after a single dose of VV^dnTGFβmm^ (Fig. 7, A and C). When treating with VV^ctrl^, we see no CRs as this model is entirely resistant to VV^ctrl^. However, C24 treated with VV does not lead to increased IFNγ in the TME (Fig. S5 I) and as such we sought to combine VV^dnTGFβmm^ with an immunotherapy capable of elevating IFNγ. We combined VV^dnTGFβmm^ with αPD-1 (Garris et al., 2018) and found remarkable synergy. While VV^ctrl^ and αPD-1 produced modest combined benefit in C24 melanoma (Fig. 7, B and C), 67% of mice experienced CRs when αPD-1 was combined with VV^dnTGFβmm^ (Fig. 7, B and C). Indeed, we found that after VV^dnTGFβmm^ and three doses of αPD-1, T_reg_, T_conv_, and CD8^+^ cells all produced significantly more IFNγ than VV^dnTGFβmm^ +Iso (Fig. S5, I–K). In the combination treatment, we also observed a loss of PD-1+Tim3+ effector T_reg_ cells (Fig. S5 L) and an increase in TCF1 in T_reg_ cells (Fig. S5 M), which may lead to reduced suppression (Mammadli et al., 2023; Osman et al., 2021; Delacher et al., 2020). We also observed increased TCF1 in the CD8^+^ T cells (Fig. S5 N). Together, these data suggest that in a tumor model that does not experience IFNγ induction from VV alone, combining with another immunostimulatory therapy such as αPD-1 can lead to the fragility of T_reg_ cells and increase effector function.

Finally, we tested this combination therapy in an abscopal setting to determine if there were any benefits of VV^dnTGFβmm^ +αPD-1 at distant uninjected sites. Mice were given two C24 tumors on opposing flanks, one of which was treated IT with VV^dnTGFβmm^, and αPD-1 therapy was started 4 d after treatment and continued three times per week (Fig. S5 O). While we observed no increase in survival (Fig. S5 P), we did observe a slowing in growth of the uninjected lesion given αPD-1 compared with isotype control (Fig. S5 Q). This shows promise that targeted combination therapy may induce responses at distant tumor sites.

## Discussion

OVs, which can inflame and lyse tumors, promote T cell infiltration, and deliver payload to the local environment, carry the potential to immunologically activate otherwise “cold” tumors. However, despite substantial investigation and investment into OVs, clinical trials have not shown broad success apart from the initial approval of T-VEC in 2014. Recently, a trial of T-VEC in combination with pembrolizumab failed in phase Ib/III in HNSCC (Harrington et al., 2020) and in phase III in melanoma (Gogas et al., 2021), despite a promising phase II trial in melanoma and a rational combination approach designed to promote new T cell influx and enhance their activity with αPD-1 blockade (Ribas et al., 2017). A phase III clinical trial of PexaVec, a VV containing the immune-stimulatory cytokine GM-CSF, in hepatocellular carcinoma also failed due to an inability to outperform standard-of-care chemotherapy (http://ClinicalTrials.gov, NCT02562755). These clinical trial failures point to a need for a deeper understanding of the unique mechanisms of resistance to oncolytics as resistance mechanisms may not apply broadly across immunotherapies. In this study, we used paired sensitive or resistant tumors (along with an immunologically inactive melanoma model) to dissect the “common” features of OV treatment versus those that ultimately produce durable responses. In doing so, we uncovered tumor-derived resistance mechanisms paving the way for a more potent therapy.

We found no differences in the kinetics of VV or lytic ability of VV in the MEER tumor models. While we also observed no differences in the infiltration of effector T cells between the tumor types, what defined efficacy was the phenotype of the pre-existing, tumor-resident T_reg_ cells, which we found to be directly related to TGFβ in the TME. We found that in mice bearing contralateral MEER^vvS^ and MEER^vvR^, the T_reg_ cells infiltrating the sensitive tumor are primed for fragility, harboring lower surface Nrp1 expression, lower TGFβ signatures (LAP-TGFβ, GARP, and CD103 surface expression), and a higher sensitivity to IFNγ via STAT1 signaling. T_reg_ cells in MEER^vvS^ were also less activated (lower PD-1, Tim3, CD25, and CD122 expression), and these tumors had higher infiltration of effector T_conv_ and CD8^+^ cells. After OV treatment, IFNγ increases in the tumor interstitial fluid in both MEER^vvR^ and MEER^vvS^; however, in accordance with a more fragile phenotype, only MEER^vvS^ infiltrating T_reg_ cells have increased IFNγ signaling and consequent IFNγ production, consistent with previous data (Overacre-Delgoffe et al., 2017). This less-suppressive TME may allow for increased effector function of the virus-stimulated, de novo infiltrate.

TGFβ is known to support and stabilize T_reg_ cells, as well as enhance tumor progression and metastasis and act directly to suppress effector T cells and dendritic cells in the TME (Massagué, 2008). We confirmed an increase in TGFβ in the TIF of MEER^vvR^ at the steady state. We and others (Park et al., 2005; Reardon and McKay, 2007) have shown that TGFβ can directly repress IFNγ signaling in T cells and, as such, TGFβ may directly act to stabilize T_reg_ cells in resistant tumors, despite high levels of IFNγ induced with VV treatment. In support of this, we found that while when T_reg_ cells are cultured with IFNγ their suppression is reduced, when cultured in the presence of both IFNγ and TGFβ their suppression was maintained. We also found that overexpressing TGFβ1 in the sensitive MEER line was enough to render these tumors resistant to VV therapy. While our data suggest that T_reg_ cells are a major responder to TGFβ within resistant tumors, it is likely that TGFβ acts on multiple cell types within the TME. For example, at baseline, T_conv_ cells in the MEER^vvR^ have higher expression of some TGFβ related markers (GARP, Nrp1, LAP-TGFβ1; Fig. S2, J, K, and M) than in MEER^vvS^. This demonstrates that these cells are also TGFβ responsive, however not to the same extent as T_reg_ cells since these markers are not expressed to the same level in T_conv_ cells. Future work will elucidate the major responders to TGFβ and those that are the most affected by its inhibition in combination with VV^dnTGFβmm^.

These data highlight that treating a heavily immunosuppressive tumor with an OV may not alter the suppressive mechanisms of the TME. While OVs are superb for mobilizing an immune response, if the TME is suppressive, additional therapeutic measures may be needed to combat environmental immunosuppression to enable this de novo response. Our data show that only in tumors primed for T_reg_ cell fragility were therapeutic responses complete. In fact, the destabilization of T_reg_ cells through IFNγ has previously been shown to be required for αPD-1 therapy (Overacre-Delgoffe et al., 2017). As such, this may be a common thread of immunotherapy; increasing T_reg_ cell sensitivity to cytokines like IFNγ helps drive a more complete anti-tumor immune response.

TGFβ inhibition has long been a target of cancer biologists and immunologists; however, it requires balancing toxicity with potency (Teixeira et al., 2020). OVs, especially double-stranded DNA viruses such as vaccinia, serve as excellent platforms to deliver a genetic payload into the tumor; our group has previously used this method to successfully deliver a metabolic modulator (Rivadeneira et al., 2019), and T-VEC is engineered to deliver a payload of GM-CSF into the tumor (Andtbacka et al., 2015). This mechanism for delivery of a TGFβ inhibitor has two benefits over systemic administration. First, it allows for local delivery of the inhibitor. As the virus is injected into the tumor and only replicates within the tumor (Fig. S1 C), the production of the inhibitor is restricted to actively replicating virus. Second, as the inhibitor is encoded within the virus, it is only produced over the period of time the virus is present; once the virus is cleared no more inhibitor will be produced. This keeps the virus local to the tumor and temporally restricted to a short window, between day 1 and 5 after treatment. Indeed, we observed no symptoms of toxicity or long-term autoimmunity in the mice treated with VV^dnTGFβmm^.

We found that VV^dnTGFβmm^ significantly increased survival in the MEER^vvR^ as well as induced complete tumor regressions in 50% of mice. Concordant with the increase in response, we also observed phenotypic changes in the TMEs of MEER^vvR^ which aligned with those of MEER^vvS^. This included a decrease in surface Nrp1, LAP-TGFβ, and GARP, and an increase in STAT1 signaling on T_reg_ cells. We also observed increases in survival and CRs in the C24 model, which is incredibly resistant to multiple forms of immunotherapy (Najjar et al., 2019; Rivadeneira et al., 2019). By combining VV^dnTGFβmm^ with αPD-1 in the C24 model, this triple therapy (oncolytic vaccinia, dnTGFβ^mm^, and αPD-1) resulted in a striking 67% of tumors completely regressing. In fact, in an abscopal model of C24, we found that this combination was even able to significantly reduce tumor growth in the uninjected lesions compared with VV^dnTGFβmm^ + isotype control. We found in the C24 model that while these tumors had high TGFβ like MEER^vvR^, treatment with VV alone did not increase IFNγ in the tumor. Treating with αPD-1 led to increased IFNγ production by all T cells in the tumor and increased responses when combined with VV^dnTGFβmm^. This confirmed the rationale for combining therapies: oncolytic vaccinia to increase tumor-infiltrating T cells, dnTGFβ^mm^ to reduce TME suppression, and αPD-1 to improve the anti-tumor T cell response and increase intratumoral IFNγ.

It has been shown by other groups that TGFβ inhibition may have an effect on viral replication (Ilkow et al., 2015; Oh et al., 2017) while others have shown no effect (Hutzen et al., 2017). In our system, the inhibitor is produced by the virus itself and as such, replication has been initiated before the inhibitor can reach appreciable levels. Our data clearly demonstrate that despite highly potent TGFβ signaling inhibition, VV was still effective at promoting a robust antitumor response as evidenced by increased T cell infiltration, and as such we do not feel this is a major concern. As previously described, we saw no adverse effects in mice treated with dnTGFβ^mm^. Our mice were treated with one IT dose; however, patients are often given multiple doses over a period of many weeks when treated with OVs. Additional preclinical studies will ultimately evaluate the effect of a long-term dosing schedule with this inhibitor.

OV immunotherapy has immense promise to act as a potent immunologic adjuvant in cancer. However, the immunologic mechanism of action has not been rigorously studied. While the majority of prior work has focused on CD8 and conventional CD4 T cells, our study highlights the importance of suppressive populations in OV therapy. Much of the focus within OVs has been on increasing the immune stimulatory effects of the virus with such mechanisms as virally produced GM-CSF and IL-12 or a combination with checkpoint blockade. Shifting the focus of the field toward understanding the mechanisms of resistance against OVs and rationally designing combination therapies to alleviate the tolerogenic mechanisms in the TME may be more beneficial to improve responses to OVs. Further, OVs may also be a more attractive way to therapeutically target TGFβ signaling, as their local and temporally restricted delivery allows for high potency without systemic toxicity. In this way, the therapeutic efficacy of many forms of immunomodulatory therapy for cancer hindered by TGFβ may be similarly improved.

## Materials and methods

### Mice

C57/BL6 mice were obtained from Jackson Laboratories, bred in-house, and bred off-site at Charles River. Foxp3-RFP reporter mice (C57BL/6-*Foxp3*^*tm1Flv*^/J), Foxp3-Ametrine reporter (C57BL/6*.Foxp3*^*flpo-*mAmetrine^), and RAG1 knockout (C57BL/6-*Rag1*^*tm1Mom*^/J) mice were bred in-house and off-site at Charles River. *Foxp3*^*Cre-ERT2.GFP*^ × *Rosa26*^*LSL.Td.Tomato*^ mice were bred in-house. Thy1.1 mice were obtained from Jackson Laboratories. Mice used in experiments were male and female between 6 and 10 wk old at the initiation of the study. All animal work and protocols in this study were approved by the University of Pittsburgh Institutional Animal Care and Use Committee, accredited by the Association for Assessment and Accreditation of Laboratory Animal Care.

### Cell culture

Tumor experiments were conducted with MEER, C24, LLC, B16-F10, MC38, and PanCO2 tumors. The MEER tumor line is an MTE that stably expresses E6/E7 and H-ras and has been rendered resistant to anti-PD-1 therapy through serial treatment in vivo (Zandberg et al., 2021). C24 is a single-cell clone derived from a melanoma tumor from a female *Pten*^*f/f*^*Braf*^*V600E*^*Tyr*^*Cre.ER*^ mouse (Najjar et al., 2019). HeLa cells, an immortalized epithelial line, were a gift from Dr. Saumendra Sarkar, University of Pittsburgh, Pittsburgh PA, USA. MFB-B11 cells, a fibroblast line from a TGFβ^−/−^ mouse transfected with a TGFβ reporter plasmid, was a gift from Dr. Andrew Hinck (Tesseur et al., 2006). MTE-LXSN cells are an immortalized MTE line containing the EV that was used to generate the original MEER line and were a gift from Dr. Robert Ferris, University of Pittsburgh, Pittsburgh PA, USA (Hoover et al., 2007). C24, B16-F10, MC38, LLC, MFB-B11, and HeLa cells were cultured in DMEM, supplemented with 10% FBS (vol/vol). MFB-B11 cells were supplemented with the addition of hygromycin (15 μg/ml). PanCO2 cells were cultured in RPMI and supplemented with 10% FBS (vol/vol). MEER and MTE-LXSN cells were cultured in DMEM/F12 supplemented with 10% FBS (vol/vol), epidermal growth factor, insulin, transferrin, hydrocortisone, cholera toxin, and tri-iodo-tyronine (all from Sigma-Aldrich; Williams et al., 2009). MEER^vvR^ was cultured on a fibronectin (Sigma-Aldrich) coated plate. All cells were maintained at 37°C with 5% CO_2_.

### Tumor models

MEER^vvR^ was generated from the MEER^vvS^ line. A MEER^vvS^ tumor (Zandberg et al., 2021) that grew out after IT treatment with 2.5 × 10^6^ PFU of VV was mechanically digested, reimplanted into C57BL/6 mice, and treated with another round of VV. This was repeated three times until a stable MEER^vvR^ line was developed.

C57BL/6 were implanted intradermally with 250,000 C24, MEER^vvR^, MEER^vvS^, MEER^vvS-EV^, or MEER^vvS-OE^ tumors. When tumors reached ∼20 mm^2^, they were treated IT with a 25-μl injection of PBS, VV/VV^ctrl^, or VV^dnTGFβmm^. VV was dosed at 2.5 × 10^5^ PFU/mouse except in Figs. 5, 6, and 7 (and corresponding supplement), which were done at 2.5 × 10^6^ PFU/mouse. For αPD-1 treatment, mice were injected intraperitoneally (IP) three times per week with αPD-1 (J43) or isotype control (Armenian Hamster IgG) at 2 mg/kg. Tumor growth was monitored until tumors reached 15 mm in any direction. In some experiments, two MEER^vvR^ or two C24 were implanted on opposing ends of the back of the same mouse. In these experiments, one tumor was treated the same as in single-tumor experiments and αPD-1 treatments occurred the same as well. In some experiments, single MEER^vvR^ tumors were treated multiple times with PBS or VV. In these experiments, mice were treated with IT PBS or VV as previously described, and at 4 and 8 d after treatment were treated again.

For tumor infiltrating lymphocyte (TIL) analysis, tumors (C24, MEER^vvS^, and MEER^vvR^) were implanted and treated the same way. At 4 or 7 d after treatment, tumors were harvested and digested as previously described (Scharping et al., 2017). In some experiments, a MEER^vvS^ and a MEER^vvR^ tumor were implanted on opposing ends of the back of the same mouse. These tumors were not treated but instead harvested at an average size of 75 mm^2^, or about 12 d after implantation.

For in vivo viral imaging, tumors (MEER^vvS^ and MEER^vvR^) were implanted and treated with VV at 2.5 × 10^5^ PFU per mouse. Starting at 24 h after treatment, mice were given an IP dose of luciferin (30 mg/ml). After 10 min, mice were imaged in the IVIS Spectrum In Vivo Imaging System. Mice were imaged every day for 5 d after treatment.

To harvest tumor interstitial fluid, tumors (MEER^vvS^, MEER^vvR^, C24, LLC, B16, MC38, and PanCO2) were harvested at ∼75 mm^2^ into an empty 15-ml conical tube. Tumors were then placed onto a 5-μm nylon membrane filter in a fresh conical tube and spun at 3,000 RPM for 20 min. The volume of interstitial fluid was recorded and then stored at −80°C until analysis via IFNγ ELISA, Luminex, or TGFβ reporter assay.

### Generating TGFβ overexpressing MEER line

The lentiviral vectors pHIV-RFP720 and pHIV-RFP720-E2A-mTGFβ were produced using the Addgene plasmid pHIV-iRFP720-E2A-Luc (#104587; Addgene) as backbone. For the pHIV-RFP720 vector, two segments of the original plasmid were produced by PCR designed to produce overhangs. Then the segments were used for a HiFi DNA Assembly (NEB). The pHIV-RPF720-E2A-mTGFβ vector was produced using a segment from the original vector produced with PCR and a gBlock from IDT. These plasmid DNA fragments were used for a HiFi DNA Assembly. Briefly, the PCR fragments were digested with DpnI (#R0176S; NEB) to remove the original plasmid without overhangs and gel isolated (#T1020S; NEB Monarch DNA Gel extraction kit). 25 ng of each DNA fragment was added into a reaction with HiFi DNA Assembly (#E2621S; NEB), and the resulting plasmid was transformed into 5-α competent *Escherichia coli* (NEB). The colonies were mini-prepped (#27104; QIAGEN) and sent for Sanger Sequencing to Genewiz to confirm. The bacteria carrying the desired plasmid was then isolated with NucleoBond Xtra Midi Plus EF (#740422; Takara).

After plasmid generation, LentiX 293T cells (#632180; Takara) were transfected with three plasmids: the viral plasmid (pHIV-RFP720 or pHIV-RFP720-E2A-mTGFβ), psPAX2 (#12260; Addgene), and pMD2.G (#12259; Addgene) at 4:3:1 ratio and the transfection reagent Xfect (#631318; Takara). After 4 h, the transfection mix was removed and fresh media was added. Viral supernatants were collected after 2 d and filtered. This fresh virus was used to spin-transduce MEER^vvS^ cells at 10% confluency for 2 h at 2,000 × *g* with 1:2,000 polybrene (stock solution 10 mg/ml). The cells were allowed to rest overnight; viral media was removed in the morning and fresh media was added. After 2 d, transfected cells were sorted by RFP720 expression. TGFβ overexpression was then confirmed by Western blot.

### OV production

VV^dnTGFbmm^ was generated as previously described in Rivadeneira et al. (2019). In brief, a Western Reserve strain of VV lacking B18R and with TK inactivation (Kirn et al., 2007) was used. The pSC65 cloning plasmid (provided by Professor Bernie Moss, National Institutes of Health) was remade using Gibson cloning (New England Biolabs) so that firefly luciferase was expressed from the pSE/L promoter and the dnTGFβmm with an IL-2 signal sequence was expressed from the p7.5 early/late promoter. This was recombined into the TK gene using Gibson cloning (NEB).

The dnTGFβmm was developed and produced in the lab of A.P. Hinck. It is described in Ludwig et al. (2021). In brief, this is mini-monomer binds TGFβRII with the same affinity as TGFβ1 and TGFβ3 dimers; however, it cannot recruit TGFβRI.

HeLa cells were infected with VV^ctrl^ or VV^dnTGFβmm^ at an MOI of 0.1 for 2 h in DMEM supplemented with 10% FBS (vol/vol). Virus was then removed and fresh media was added. Once cytopathic effect was visible (∼48 h after infection), cells were harvested into their supernatant by gentle rinsing. Cells were then pelleted, resuspended into 10 ml of 10 mM Tris-HCl (pH 7.0), and lysed by three cycles of freeze–thaw. The resulting supernatant was layered onto a sucrose cushion (36% sucrose in 10 mM Tris-HCl, pH 7.0) and spun for 2 h at 14,500 RPM at 4°C. The viral pellet was resuspended in 200 μl of 10 mM Tris-HCl and stored at −80°C. Purified virus was tittered on HeLa cells using a crystal violet plaque assay.

### Flow cytometry

Cell surface staining was performed on ice in PBS for 20 min with surface antibodies and Zombie Viability Dye (BioLegend). Cells were then washed in PBS and either run for live panels or fixed with 4% paraformaldehyde for 5 min at room temperature and then washed in PBS. For phospho staining, cells were fixed with ice-cold 90% methanol at −20°C for 20 min, then washed with permeabilization wash buffer (eBioscience), and stained overnight at 4°C. For nuclear staining, cells were fixed with the Foxp3 Transcription factor fixation kit (eBioscience) for 20 min at room temperature and then stained overnight at 4°C. For cytokine restimulation, lymphocytes were restimulated with PMA and ionomycin for 5.5 h in the presence of a Golgi plug (BD Biosciences). Surface staining was performed as above followed by fixation with the CytoFix/CytoPerm kit (BD Biosciences) and stained overnight at 4°C for intracellular cytokine staining.

### Flow antibody information

The following antibody clones were utilized for flow cytometry experiments: CD4 (GK1.5; BioLegend), CD8 (53-6.7; BioLegend), Nrp1 (3E12; BioLegend), IFNγ Receptor 1 (2E2; eBioscience), IFNγ Receptor β chain (MOB-47; BioLegend), PD-1 (29F.1A12; BioLegend), Tim3 (RMT3-23; BioLegend), CD45 (30-F11; Biolegend), LAP-TGFβ1 (TW7-16B4; BioLegend), GARP (F011-5; BioLegend), CD103 (2E7; BioLegend), TCF1/7 (812145; R&D Systems), CD44 (IM7; BioLegend), CD62L (MEL-14; BioLegend), CD25 (PC61; BioLegend), CD122 (TM-β1; BioLegend), Granzyme B (GB11; BioLegend), Foxp3 (FJK-16 s; eBioscience), TNFa (MP6-XT22; BioLegend), IFNγ (XMG1.2; BioLegend), pSTAT1 ser727 (A15158B; BioLegend), and pSTAT1 tyr701 (A17012A; BioLegend).

### Bulk RNA-seq analysis

MEER^vvS^ and MEER^vvR^ were cultured in vitro as described above, and cDNA was generated using SMART-seq HT kit (Cat # 634456; Clontech) using 1,000 cells. cDNA product was checked by Tape Station D5000 from Agilent Technologies 2200 to make sure cDNA was successfully generated. Library construction was done using Nextrera XT kit # 15031942 from Illumina. 1 ng cDNA was used in a total volume 5 µl. Sequencing was done using NextSeq 500 System. High Output 75 Cycles kit with run Parameter Paired Read 150 cycles (2 × 75).

Sequencing reads were trimmed for adapters and then aligned to *Mus musculus* reference genome (Mus_musculus_ensembl_v80_Sequence) using CLC Genomics Workbench. Using CLC Genomics Workbench, differential gene expression was found with statistical cut-offs of P value <0.05, max group means >1, and |foldchange| >1.5. Heatmaps were generated using R package pheatmap with log_2_ transformed transcript counts per million (trimmed mean of M-values adjusted).

Gene set enrichment analysis (Mootha et al., 2003; Subramanian et al., 2005) was performed on differentially expressed genes using the Hallmark gene set.

### ELISA

The ELISA plate was coated with 50 μl of capture antibody (Anti-Mouse IFNγ; BioLegend; 1:1,000 in PBS) at 4°C overnight. The next morning the plate was washed three times with wash buffer (PBS + 0.05% Tween-20). The plate was blocked with 200 μl blocking buffer (PBS + 1% BSA) for 1 h at room temperature. After washing three times, the samples and standard curve were added (50 μl), diluted in blocking buffer, and incubated at room temp for 1 h. The plate was washed five times and the detection antibody was added (Biotin Anti-Mouse IFNγ; BioLegend; 1:1,000 in blocking buffer) for 1 h at room temperature After five washes, HRP streptavidin (1:500 in blocking buffer; BioLegend) was added and incubated for 30 min at room temperature After seven washes, tumor mutational burden substrate A (50 μl) and tumor mutational burden substrate B (50 μl) were added to develop the samples, and 50 μl of H_2_SO_4_ was added to stop the reaction. The plate was read at 450 nm.

### TGFβ reporter assay and IC50

The reporter assay protocol was followed from Tesseur et al. (2006). Briefly, MFB-B11 cells were plated at 40,000/well in a flat-bottomed 96-well plate and allowed to adhere for 3–4 h. Standards and samples were then added in 50 μl of DMEM + 10% FBS. After 24 h, 20 μl of supernatant was pulled and added to a 96-well ELISA plate with 20 μl of p-nitrophenyl phosphate. Samples were incubated for 4 h at room temp in the dark and then the absorbance was read at 405 nm.

The inhibitory activity (IC50) of the dnTGFβmm was determined using the protocol previously described in Kim et al. (2017). Briefly, HEK293 cells stably transfected with the CAGA12TGFβ reporter were used for the luciferase reporter assays. Cells were cultured with the dnTGFβmm in a dose-response gradient and stimulated with a sub-EC50 dose of TGFβ3 (10 pm) for 16 h. Cells were trypsinized, luciferase activity was read with a Promega GloMax luminometer (Promega), and luciferase activity was normalized to total protein levels determined by bicinchoninic acid protein assay. GraphPad Prism 6 was used to fit the data to standard models for ligand activity (EC50) and ligand inhibitory activity (IC50; GraphPad).

### Immunoblotting

T_reg_ cells were sorted from spleen and lymph nodes of Foxp3^*RFP*^ or Foxp3^*Ametrine*^ mice by Foxp3-reporter expression (MA900; Sony) and then 250,000 cells per condition were cultured in serum-free media for 3 h followed by culture in 100 ng/ml IFNγ (Peprotech), 100 ng/ml TGFβ (R&D systems), or both for 30 min. Cells were then harvested for Western blotting.

MEER^vvR^, MEER^vvS^, and MTE-LXSN cells were cultured at 50,000 per well of a 24-well plate. 100,000 cells were harvested 24–248 h later for lysis.

Cells were lysed in 1% NP-40 lysis buffer, cell debris was removed by centrifugation, and samples were boiled for 10 min in NuPAGE LDS sample buffer (Thermo Fisher Scientific) and dithiolthreitol (Thermo Fisher Scientific). Lysates were then separated by SDS-PAGE using 4–12% Bio-Rad gels. Samples were then transferred from the gel to a polyvinylidene difluoride membrane and blocked in 5% milk in Tris-buffered saline 0.1% Tween-20 (TBST). The membrane was then incubated overnight at 4°C with primary antibodies diluted in a blocking buffer. The membrane was washed five times for 5 min in TBST. The membrane was incubated with secondary antibody (anti-mouse or anti-rabbit HRP, Jackson ImmunoResearch) in blocking buffer for 1 h at room temperature and subsequently washed five times for 5 min with TBST. Protein was visualized by chemiluminescence by using Western Lightning ECL (PerkinElmer). Antibodies were obtained from the following companies: STAT1 (Cell Signaling), pSTAT1 Y701 (Cell Signaling), β-actin (Santa Cruz), pSMAD2 (Cell Signaling), SMAD2 (Cell Signaling), Ras G12V (Cell Signaling), and p53 (Cell Signaling).

### Microsuppression assay

T_reg_ cells were sorted from spleen and lymph nodes of Foxp3^*RFP*^ or Foxp3^*Ametrine*^ mice by Foxp3-reporter expression (MA900; Sony), then spun down and sorted again to ensure purity. Cells were then stimulated overnight with PMA (0.1 µg/ml), ionomycin (1 µg/ml), and IL2 (100 U/ml) at 1 × 10^6^ cells/ml. The next morning, stimulation was washed off and cells were placed into no cytokine, IFNγ (100 ng/ml), TGFβ (100 ng/ml), or both, with IL2 (100 U/ml). After 48 h, T_reg_ cells were sorted from their conditions by Foxp3-reporter. CD4^+^ responder cells and APCs were also sorted from a Thy1.1+ mouse. CD4^+^ responder cells were stained with Cell Trace Violet proliferation dye (Thermo Fisher Scientific). CD4^+^ responder cells were then mixed 1:1 with APCs and αCD3 (3 μg/ml; BioLegend). 30k T_reg_ cells were plated in the top row of a 96-well round bottom plate and serially diluted. The APC:responder mix was added into these wells so that the first well contained a 1:2 T_reg_:responder ratio and that was diluted by half at each dilution. After 72 h, the cells were analyzed by live flow cytometry to read out the proliferation of the CD4^+^ Thy1.1+ responder cells compared with no T_reg_ cell control.

### Online supplemental material

The supplementary information contains data confirming similar viral kinetics in the MEER^vvR^ and MEER^vvS^ tumors (Fig. S1) as well as additional dLN flow data and profiling of T_conv_ cells in the tumor (Figs. S2, S3, and S4). It also contains confirmatory data from the generation of the MEER^TGFb-OE^ tumor line (Fig. S4 A) as well as additional profiling and tumor growth data from the melanoma model C24 (Fig. S5).

## Supplementary Material

SourceData F4 is the source file for Fig. 4.

SourceData F5 is the source file for Fig. 5.

SourceData FS1 is the source file for Fig. S1.

SourceData FS4 is the source file for Fig. S4.

## Data Availability

The data underlying Fig. S1 G are openly available in GEO at GSE188506 (oncolytic virus-mediated delivery of a potent TGFβ inhibitor overcomes therapeutic resistance driven by a tolerogenic TME [bulk RNA-seq]).
