# Supplementary figures and images for: An oncolytic virus–delivered TGFβ inhibitor overcomes the immunosuppressive tumor microenvironment
Source: J Exp Med. 2023 Aug 8;220(10):e20230053. doi: 10.1084/jem.20230053 (PMC10407786; doi:10.1084/jem.20230053)

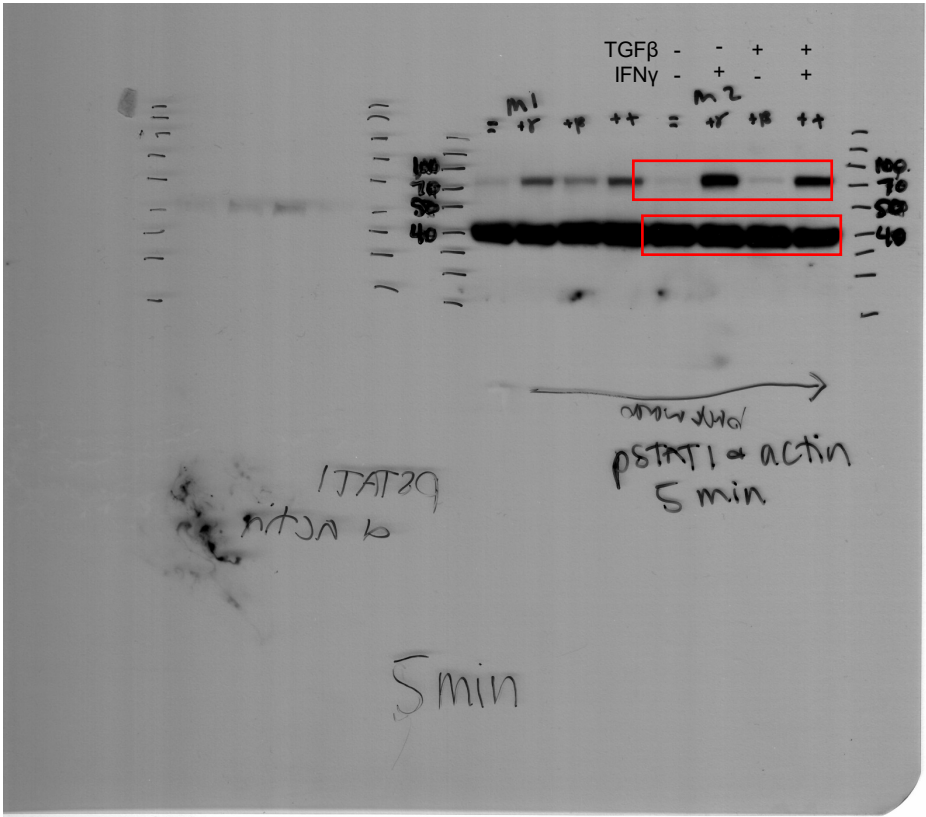

pSTAT1

B-actin

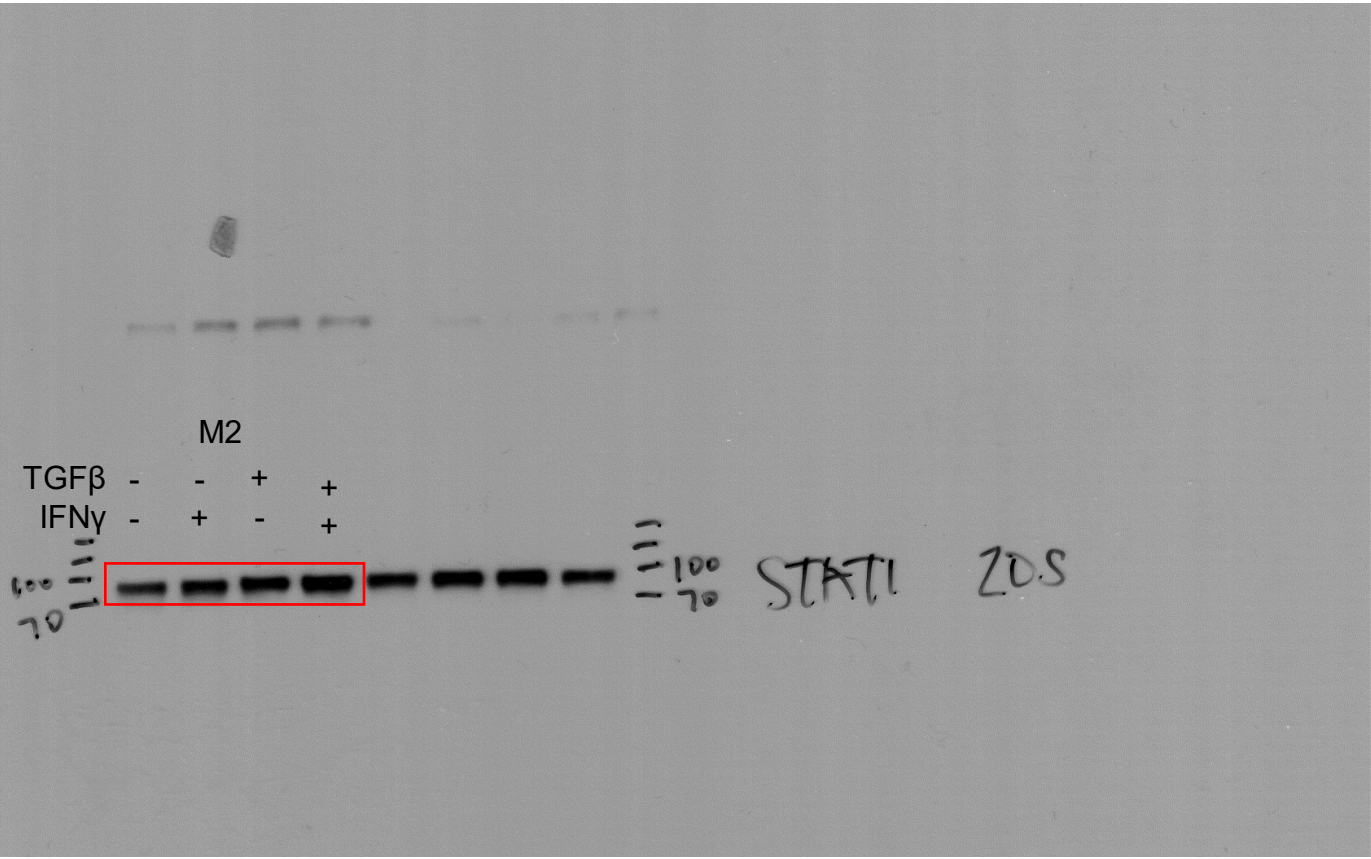

Supplement: SourceData F4 — is the source file for Fig. 4. [file JEM_20230053_SourceDataF4.pdf]

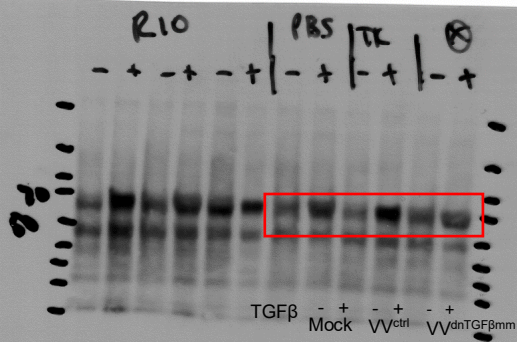

pSMAD2 T cell  
5 min

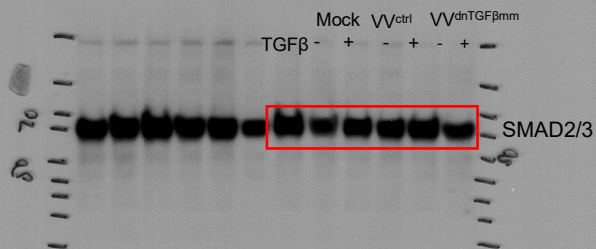

T cell  
total  
SMAD  
1 min

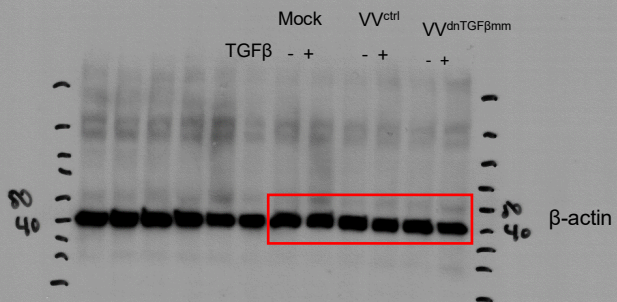

actin  
T cells

1 min

Supplement: SourceData F5 — is the source file for Fig. 5. [file JEM_20230053_SourceDataF5.pdf]

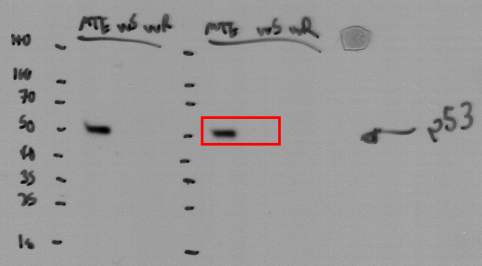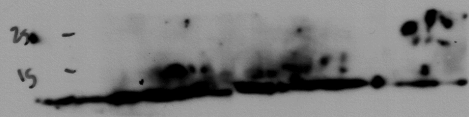

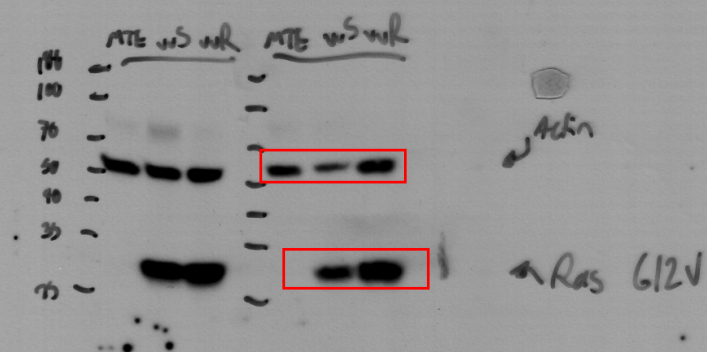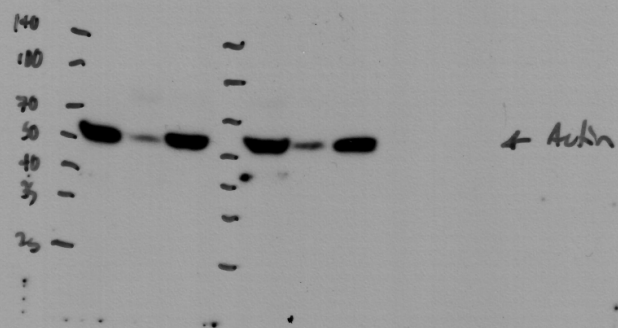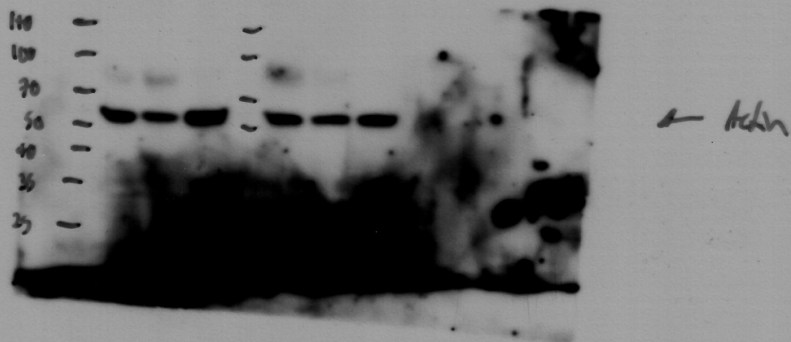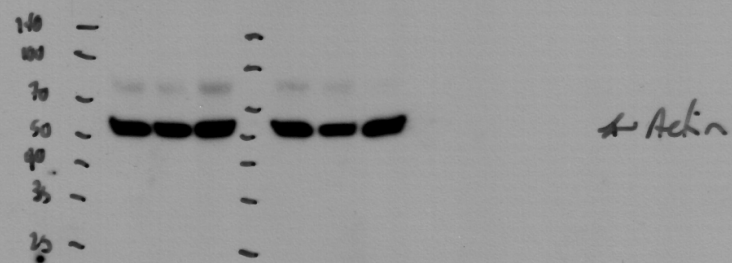

Supplement: SourceData FS1 — is the source file for Fig. S1. [file JEM_20230053_SourceDataFS1.pdf]

2min  
TGF $\beta$   
(back)

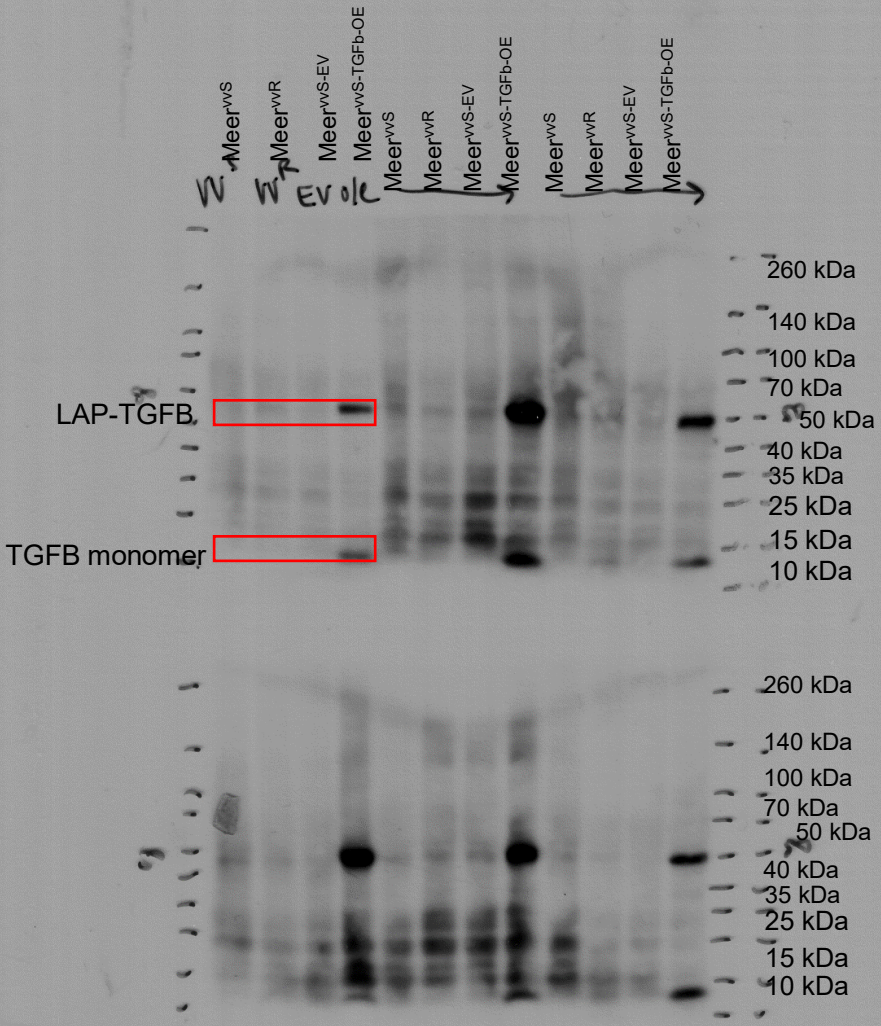

Im. actin

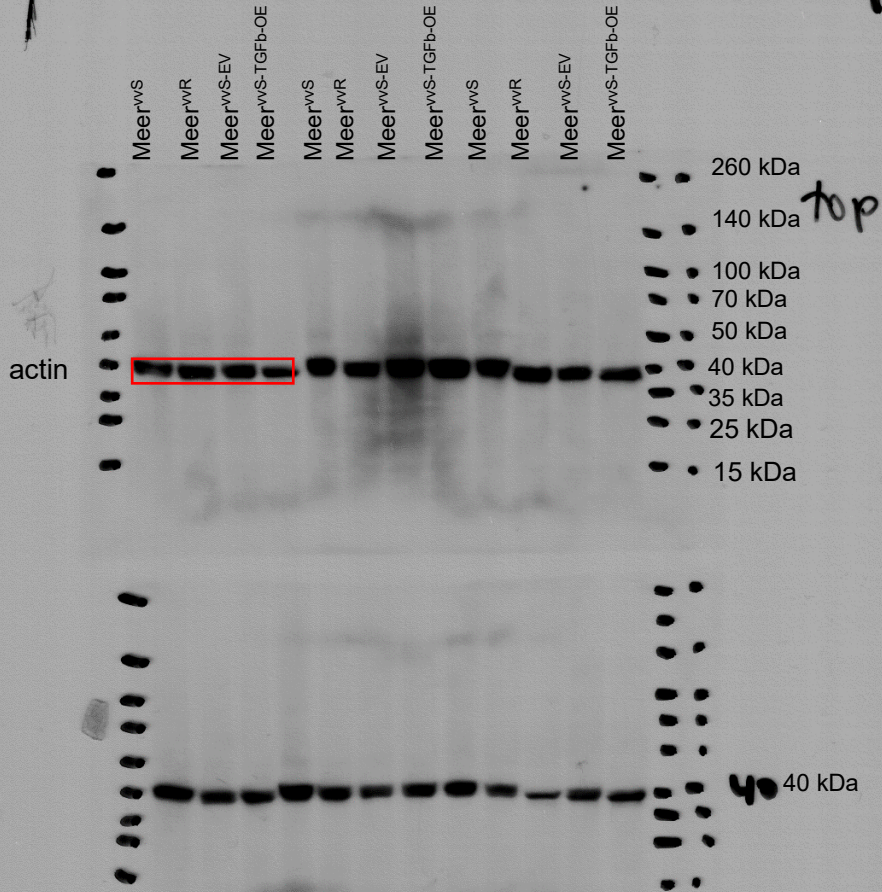

Supplement: SourceData FS4 — is the source file for Fig. S4. [file JEM_20230053_SourceDataFS4.pdf]
